# Reusable magnetite nanoparticles–biochar composites for the efficient removal of chromate from water

**DOI:** 10.1038/s41598-020-75924-7

**Published:** 2020-11-04

**Authors:** Md. Samrat Alam, Brendan Bishop, Ning Chen, Salman Safari, Viola Warter, James M. Byrne, Tyler Warchola, Andreas Kappler, Kurt O. Konhauser, Daniel S. Alessi

**Affiliations:** 1 Department of Earth and Atmospheric Sciences, University of Alberta, 1-26 Earth Sciences Building, Alberta, T6G 2E3 Canada; 2 Canadian Light Source Inc., University of Saskatchewan, 114 Science Place, Saskatoon, SK S7N 0X4 Canada; 3 Geomicrobiology, Center for Applied Geoscience, University of Tübingen, 72076 Tübingen, Germany; 4 Present Address: Department of Earth Sciences, University of Toronto, 22 Russell Street, Toronto, ON M5S 3B1 Canada; 5 Present Address: Department of Geology, University of Regina, 3737 Wascana Parkway, Regina, SK S4S 0A2 Canada

**Keywords:** Environmental sciences, Geochemistry

## Abstract

Biochar (BC) and magnetite (Fe_3_O_4_) nanoparticles (MNP) have both received considerable recent attention in part due to their potential use in water treatment. While both are effective independently in the removal of a range of anionic metals from aqueous solution, the efficacy of these materials is reduced considerably at neutral pH due to decreased metal adsorption and MNP aggregation. In addition to synthetic metal oxide–biochar composites for use in treatment and remediation technologies, aggregates may also occur in nature when pyrolytic carbon is deposited in soils. In this study, we tested whether magnetite synthesized in the presence of biochar leads to increased removal efficiency of hexavalent chromium, Cr(VI), at the mildly acidic to neutral pH values characteristic of most natural and contaminated aqueous environments. To do so, magnetite nanoparticles and biochar produced from ground willow were synthesized to form composites (MNP–BC). Batch studies showed that MNP–BC markedly enhanced both adsorption and reduction of Cr(VI) from aqueous solution at acidic to neutral pH as compared to MNP and BC separately, suggesting a strong synergetic effect of hybridizing Fe_3_O_4_ with BC. Mechanistically, the Cr(VI) removal processes occurred through both adsorption and intraparticle diffusion followed by reduction to Cr(III). Synchrotron-based X-ray absorption spectroscopy analyses confirmed that Cr(VI) was reduced at the surface of MNP–BC, with electrons derived directly from both biochar and magnetite at low pH, while at near-neutral pH, biochar increased Cr(VI) reduction by inhibiting MNP aggregation. Extended X-ray absorption fine structure fitting results confirmed that the Cr(III) precipitates consist of Cr(OH)_3_ and chromite (Cr_2_FeO_4_) nanoparticles. Our results demonstrate that MNP–BC composites have great potential as a material for the treatment of chromate-containing aqueous solutions across a wide range of pH values, and provide information valuable broadly relevant to soils and sediments that contain biochar.

## Introduction

Iron(II)-bearing minerals have received considerable attention for the reductive immobilization of water contaminated with redox-active metals^1,2^. In particular, Fe(II) is an effective reductant of Cr(VI), either in the form of bichromate (HCrO_4_^−^ at acidic pH) or chromate (CrO_4_^2−^ at alkaline pH)^3^. Among Fe(II)-containing materials, magnetite (Fe_3_O_4_) nanoparticles (MNP) are attractive for remediation applications because they are inexpensive to produce, recyclable, easily separable from magnetite containing admixtures, and possess high surface area^4–5^. The Fe(II) in magnetite can reduce Cr(VI) to Cr(III), producing Cr(III) precipitates such as Cr(OH)_3_ and Fe_x_Cr_1−x_(OH)_3_ in aqueous solutions^6–7^. However, the reaction kinetics of Cr(VI) and Fe(II) in MNPs is strongly dependent on solution pH and the Fe(II)/Fe(III) stoichiometry in magnetite^6–7^^.^ Magnetite alone is not effective under near-neutral and basic conditions, and the Fe(II) in magnetite is highly susceptible to oxidation, leading to the formation of Fe(III) materials^8–12^ Under neutral to basic pH conditions, MNPs aggregate, resulting in a dramatic diminishment of their metal adsorption and/or reduction capacity^3,8–9^. For instance, Peterson et al.^13^ showed that the reduction reaction of Cr(VI) at the surface of MNPs was passivated at pH 7 due to the aforementioned aggregation and MNPs are negatively charged at high pH which repels anionic species, demonstrating that MNPs alone have little capacity to reduce Cr(VI) at neutral pH.

Carbon-based functional materials have also received considerable attention in the field of water purification because of their high surface area, electrical conductivity and chemical stability^14,15^. Biochar (BC), a 3D porous carbonaceous material, can be produced by the pyrolysis of carbon-rich biomass such as crop residue, manure and solid waste, under limited oxygen conditions^16–17^. Biochar has been proven effective at removing metals and organics from aqueous solution due to its pore-rich structure, oxygen-containing functional groups and elevated cation exchange capacity^16–17^, and is cost-effective compared to activated carbon and graphene oxides^18^. While the adsorption capacity of BC for metals has been well studied^16–17,19–20^, BC-mediated redox processes are far less studied. A few studies show that BC can donate, accept and transfer electrons via either biotic or abiotic pathways^21–27^. For example, Kappler et al*.*^23^ and Wu et al*.*^24^ demonstrated that BC enhanced the microbial reduction of ferrihydrite (Fe(OH)_3_) and hematite (α-Fe_2_O_3_) by functioning as an electron shuttle. More recently, Rajapaksha et al*.*^28^ and Cossio^29^ showed direct evidence of Cr(VI) reduction to Cr(III) by BC using synchrotron-based X-ray absorption spectroscopy (XAS). Cossio^29^ further showed that Cr(VI) reduction by BC depends on solution pH, and that it is essentially unable to reduce Cr(VI) once solution pH is below approximately 4. A deeper understanding of the kinetics of redox reactions involving BC at a range of pH values is still required to enhance its application in treating redox-sensitive metals.

Organic–inorganic composites and hybrid materials for reductive immobilization of Cr(VI) are less well-investigated, owing to the difficulties in achieving a synchronous reduction and adsorption efficiency^30–33^. Jiang et al*.*^34^ and Wang et al*.*^35^ successfully employed magnetite coated in humic acid and m-phenylenediamine to efficiently remove Cr(VI) from solution. While effective, the application of these methods could be challenging due to their complicated and costly nature. Although a few studies have demonstrated that BC can be used as a substrate to support zero valent iron and magnetite for enhanced removal of metals from solution, including Cr(VI)^25–27,36–38^, the mechanisms underlying Cr(VI) reduction and the kinetics of the redox reactions are yet to be well characterized^36–38^. The depositing of MNPs onto BC is promising because the latter is a considerably less expensive substrate than other carbonaceous materials, such as activated carbon or graphene. MNPs have excellent magnetic properties, stability, wide availability, and are not toxic to aquatic life^1–2,39–40^. One of the many advantages of magnetite nanoparticles is that their separation from aqueous solution can be achieved using magnetic fields. The separation process responds to the magnetic moments of the nanoparticles—the higher the magnetic moments, the faster the separation processes^41–42^. Coupling MNPs with BC also increases the effective surface area of the MNPs by preventing their agglomeration and concomitant passivation of reactivity that is shown to occur at near-neutral and basic pH levels^34–35^.

Here we demonstrate the efficacy of composites of MNPs and BC to enhance the adsorption and reduction of Cr(VI) across a wide range of water chemistries (i.e., across acidic to neutral pH and in the presence of oxidants). The objectives of the study were to: (1) gain a mechanistic understanding of the interaction (adsorption and reduction) of Cr(VI) onto the surfaces of MNP–BC composites; (2) determine the effects of pH and oxidants on Cr(VI) removal and the kinetics of Cr(VI) adsorption and reduction; and (3) characterize the products of Cr(VI) reduction that form on the composite surface. Results from high resolution electron microscopy and X-ray absorption spectroscopy (XAS) analyses allowed us to develop a better understanding of the molecular scale reaction mechanisms of Cr(VI) onto the surface of the composites. The resulting composite is highly efficient, reusable and may be a cost-effective sorbent for the reductive immobilization of chromate from aqueous solution. The development of a molecular scale understanding of the adsorption and reduction processes at the surface of MNP–BC aggregates provides valuable information to both Cr(VI) contaminant immobilization and processes relevant to soils and sediments that contain pyrogenic carbon.

## Results and discussion

### Characterization of adsorbents

**Figure 1:**
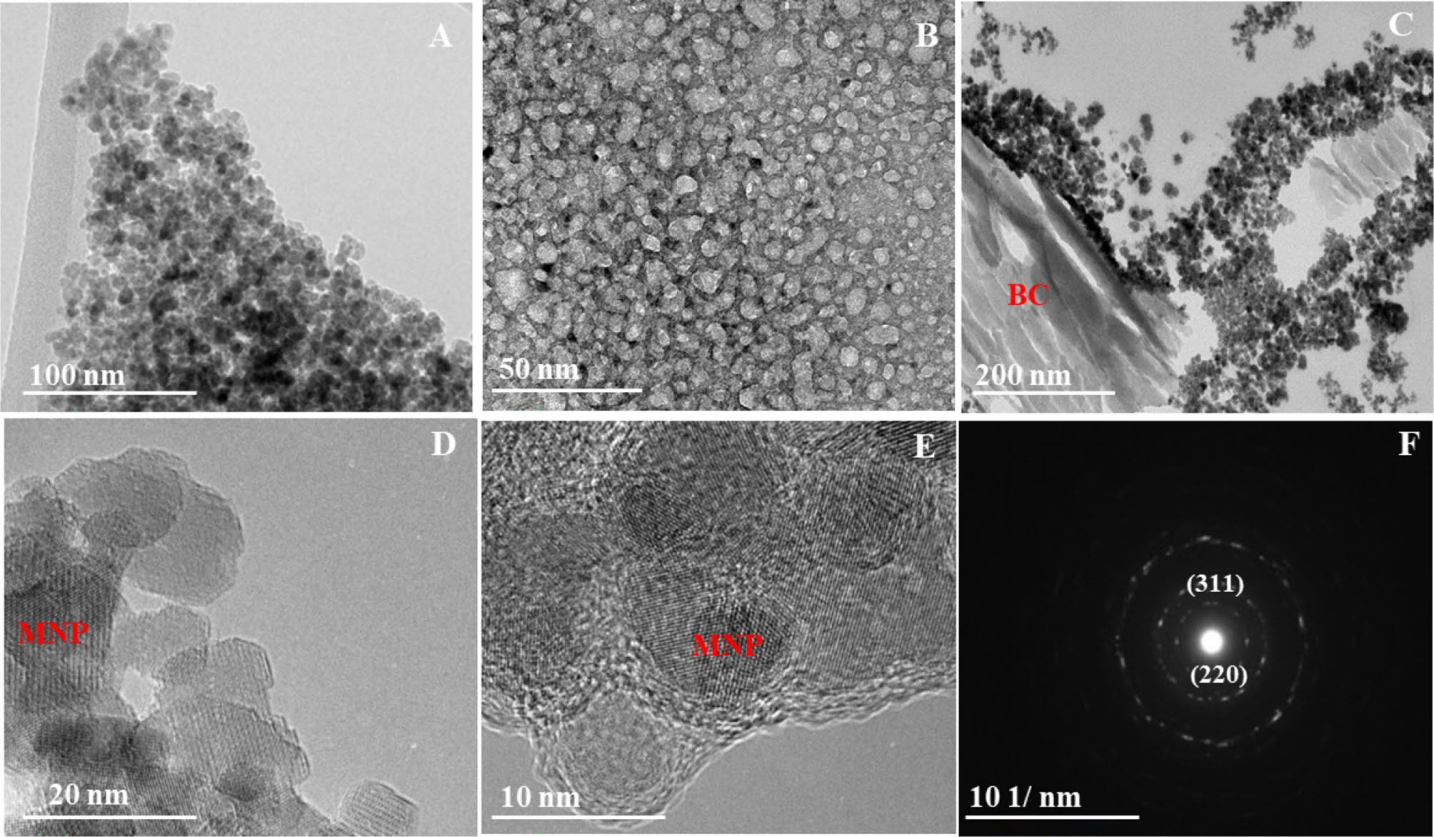
HR-TEM images of (**A**,**B**) MNPs; (**C**) MNP–BC (10 mM Fe concentration); (**D**,**E**) MNP–BC (2 mM Fe concentration); and (**F**) SAED pattern of MNP–BC.

The elemental analysis of BC showed that it is primarily composed of C (76 wt%) and O (10 wt%). The specific surface area of BC as measured by Brunauer–Emmet–Teller (BET) is 319 m^2^/g (Table S2). BC is conductive; the sheet resistance of BC decreased as BC concentration increased (Fig. S1). TEM and HRTEM-SAED images (Fig. 1) of MNP–BC show crystalline magnetite nanoparticles in the composites at various MNP to BC weight ratios. The MNP particles exhibited generally irregular and rounded morphologies, with a size range between approximately 10–20 nm, and in agreement with Fe_3_O_4_, ferrozine^43^ results showed Fe(II)/Fe(III) ratios of approximately 0.5. The particle sizes were estimated using the TEM scale bars by measuring a number of particles (n = 15–20). DLS analysis showed that size ranges of MNPs increased with increasing pH (Fig. S2); this is likely attributed to their agglomeration at higher pH, as shown in previous studies^5–7^. SEM images of MNP–BC composites (Fig. S3) showed MNPs are widely distributed on the surface of BC, evidencing that BC prevents MNP agglomeration.

FT-IR spectra (Fig. S4A) of MNPs and MNP–BC show a distinct band at 594 cm^−1^ that can be ascribed to Fe–O groups, consistent with the presence of magnetite^44^. The spectra also showed stretching vibrations of water molecules (free O–H) of MNPs and MNP–BC at 3210 cm^−1^ and 1410 cm^−1^, respectively. BC has strong bands at 3425 cm^−1^ and 1590 cm^−1^, corresponding to –OH and –C=O groups^22,45^, respectively. The same bands are observed in the MNP–BC composite. The vibrational band at 1110 cm^−1^ is assigned to stretching of the C–O–C moiety.

Zeta potential measurements, an indicator of surface charge, versus pH (Fig. S4B) of MNPs, MNP–BC and BC showed that the isoelectric points—the pH values at which the particles carry no net electrical charge—for MNP and MNP–BC are near pH 6 and pH 5, respectively, whereas BC has an isoelectric point near pH 2. This demonstrates that the presence of organic functional groups slightly lowers the isoelectric point of the magnetite. XPS analyses (Fig. S4C,D) of MNPs and MNP–BC further showed that the Fe2p_3/2_ peak is centered at 711.1 eV, a result which can be ascribed to the convolution of both Fe(II) and Fe(III) signals from Fe_3_O_4_^46^_._ XPS spectra (Fig. S4E,F) are also consistent with the presence –C=O groups in both MNP–BC and BC. Organic functional groups (e.g., carboxyl), such as those present on humic substances, confer a negative charge which has the effect of lowering the overall surface charge of magnetite and decreases its isoelectric point (IEP)^39^. XRD analyses confirmed that MNPs and MNP–BC contain Fe_3_O_4_ at various time intervals after synthesis and also after exposure to atmospheric conditions, i.e., exposure to O_2_ (Fig. S5). Our XRD patterns show diffraction peaks at 2θ = 35.42° and 43.05°, which correspond to the (220) and (311) planes and represent the face-centered cubic inverse spinal structure of crystalline Fe_3_O_4_^12^. The deviation in plane 400 at 43.05° over time can be attributed to a minor transformation of magnetite to maghemite, but we did not observe diffraction peaks consistent with maghemite. In addition to magnetite, the reflection of quartz is also observed in MNP–BC.

The potentiometric titration data were modeled using a non-electrostatic, discrete site surface complexation model to determine the proton binding constants and corresponding site concentrations for reactive surface sites of BC, MNPs and MNP–BC. A model that uses three sites for BC and MNP–BC and one amphoteric site for MNPs yielded the best fits to the experimental data for each sorbent, where variance, V(Y), values were in the range of 0.1 < V(Y) < 20^47^ (Fig. S6). The pK_a_ values for site 1 in BC and MNP are approximately 2.4 to 3.0, within the range of pK_a_ values of carboxyl groups (pK_a_ = 1.7–4.7)^48–50^. The pK_a_ value for site 1 in MNPs of 5.0 corresponds to Fe–O (pK_a_ =  ~ 5). The pK_a_ values calculated for sites 2 and 3 of BC and MNP–BC, ranging between 6.3 to 9.0, correspond to lactones (pK_a_ =  ~ 6) and phenolic hydroxyl (pK_a_ =  ~ 9.5) groups (Table S3)^44,48–52^. The proton-active functional groups, including –C=O, –OH and Fe–O, that were used in modeling the potentiometric titration data were also detected in both FT-IR and XPS analyses, as is discussed above.

### Effect of pH on adsorption and reduction of Cr(VI)

**Figure 2:**
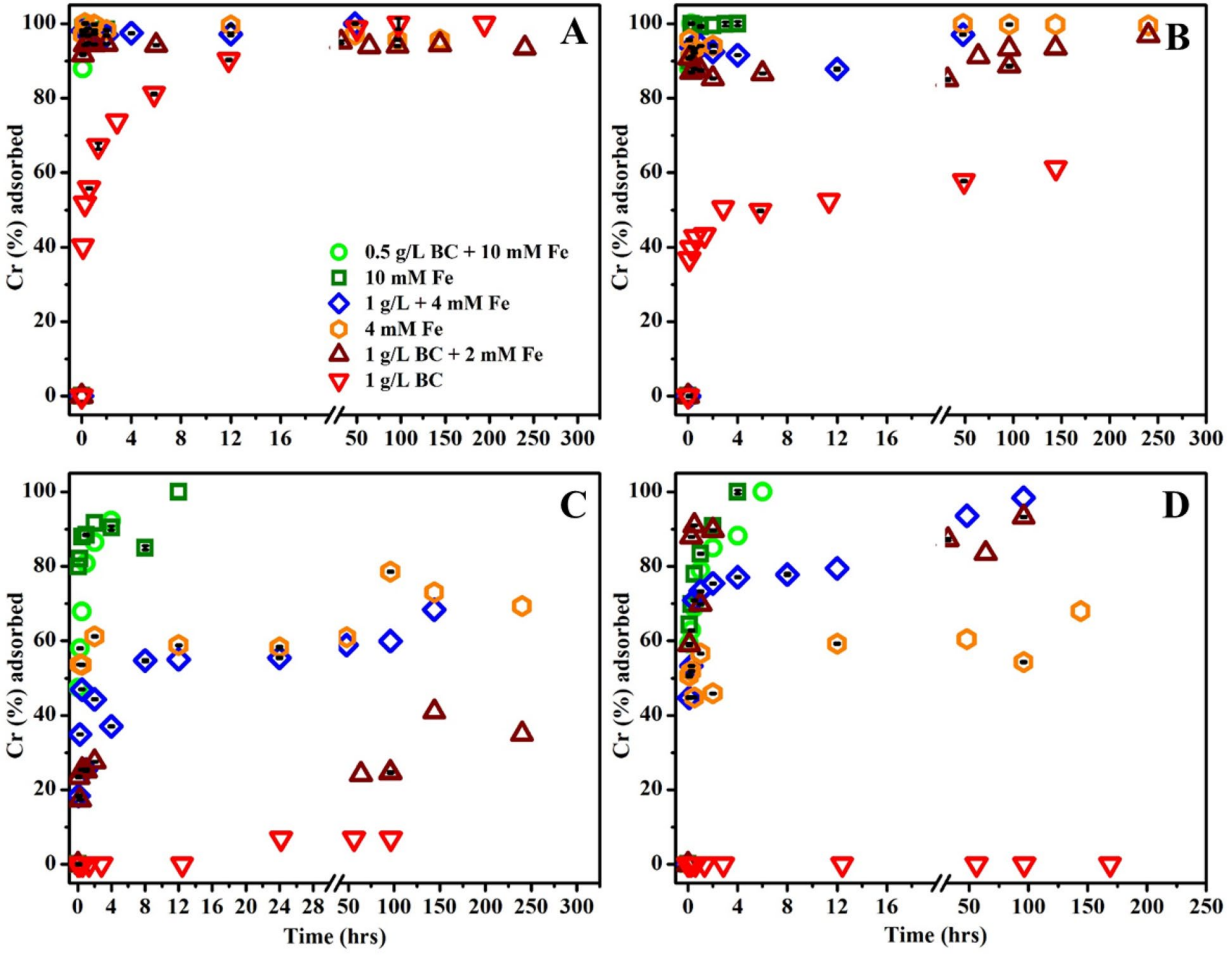
Cr(VI) adsorption by MNP–BC; MNPs and BC at: (**A**) pH 2; (**B**) pH 3; (**C**) pH 5 and (**D**) pH 7.

The pH-dependent adsorption kinetics of Cr(VI) for BC, MNPs and MNP–BC were studied in the pH range of 2–7 (Fig. 2), levels relevant to those found from acid mine drainage (AMD) and natural waters associated with rivers and groundwater. pH significantly influences the adsorption capacities of sorbents, as both the sorbent net surface charge and the aqueous speciation of Cr(VI) change with pH (Fig. S7)^35^. At the lowest pH tested (pH 2), complete removal of Cr from solution was achieved for all of sorbents (Fig. 2A). However, the experimental results showed that magnetite dissolved at pH 2 and the resulting dissolved Fe ions subsequently adsorbed to BC when the pH was adjusted to 2 in the composites. To further test the mechanism of Cr(VI) removal at pH 2, Fe^2+^ and Fe^3+^ were dissolved into a solution (without forming MNP) in the presence of BC and Cr (VI). We found that Cr(VI) was also removed from the solution completely. Hence, the results demonstrate that instead of magnetite, dissolved Fe^2+^ played the dominant role in Cr(VI) removal at pH 2. At pH 3, BC removed only 60% of the total Cr(VI) from solution, which was further reduced to 10% at pH 5, and to no measureable removal at pH 7 (Fig. 2B–D). However, MNPs and the MNP–BC composites showed higher Cr(VI) removal efficiencies of between 90 and 100% at pH 3. The adsorption capacity of MNP–BC at 4 mM Fe+ 1 g L^−1^ BC and 2 mM Fe+ 1 g L^−1^ BC was nearly 100% at pH 7, considerably higher than the 60% removal performance of MNPs at 4 mM Fe (Fig. 2D). The results clearly indicate that BC–Fe_3_O_4_ composites enhance Cr(VI) removal from solution. Zeta potential measurements also show that the isoelectric points of BC, MNP, and MNP–BC were approximately 2.2, 5.8 and 5.5, respectively (Fig. S4B), and the pH of these isoelectric points increased with Cr-loading, to 7.0, 7.8, and 3.5 for MNPs, MNP–BC and BC, respectively (Fig. S8). Above the pH of the isoelectric point, the surface of the adsorbents becomes negative. Since the most common aqueous species for Cr(VI) are CrO_4_^2−^, HCrO_4_^−^, and Cr_2_O_7_^2−^^34,53^, electrostatic repulsion between the adsorbent and adsorbate inhibits Cr(VI) adsorption to BC above pH 2 and MNPs above pH 6. However, our results showed that the repulsive electrostatic interaction between the MNP–BC and Cr(VI) species does not inhibit the removal of Cr(VI) from solution to MNP–BC even at pH 7, above the isoelectric point at pH 5. The lack of pH effects on Cr(VI) removal by MNP–BC at neutral pH suggests that adsorption is governed by surface complexation rather than electrostatic interactions^34–35^. Our results showed that MNPs are effective to adsorb Cr(VI) up to pH 5; however, the removal efficiency decreases by pH 7, likely due to aggregation of MNPs as demonstrated by the DLS measurements (Fig. S2). The results show that the Cr(VI) removal efficiency of MNP–BC, even at a lower BC loading of 1 g L^−1^ BC, was much higher at neutral pH as compared to MNP or BC alone, suggesting a positive effect of BC on the reactivity of MNPs in the composite materials.

**Figure 3:**
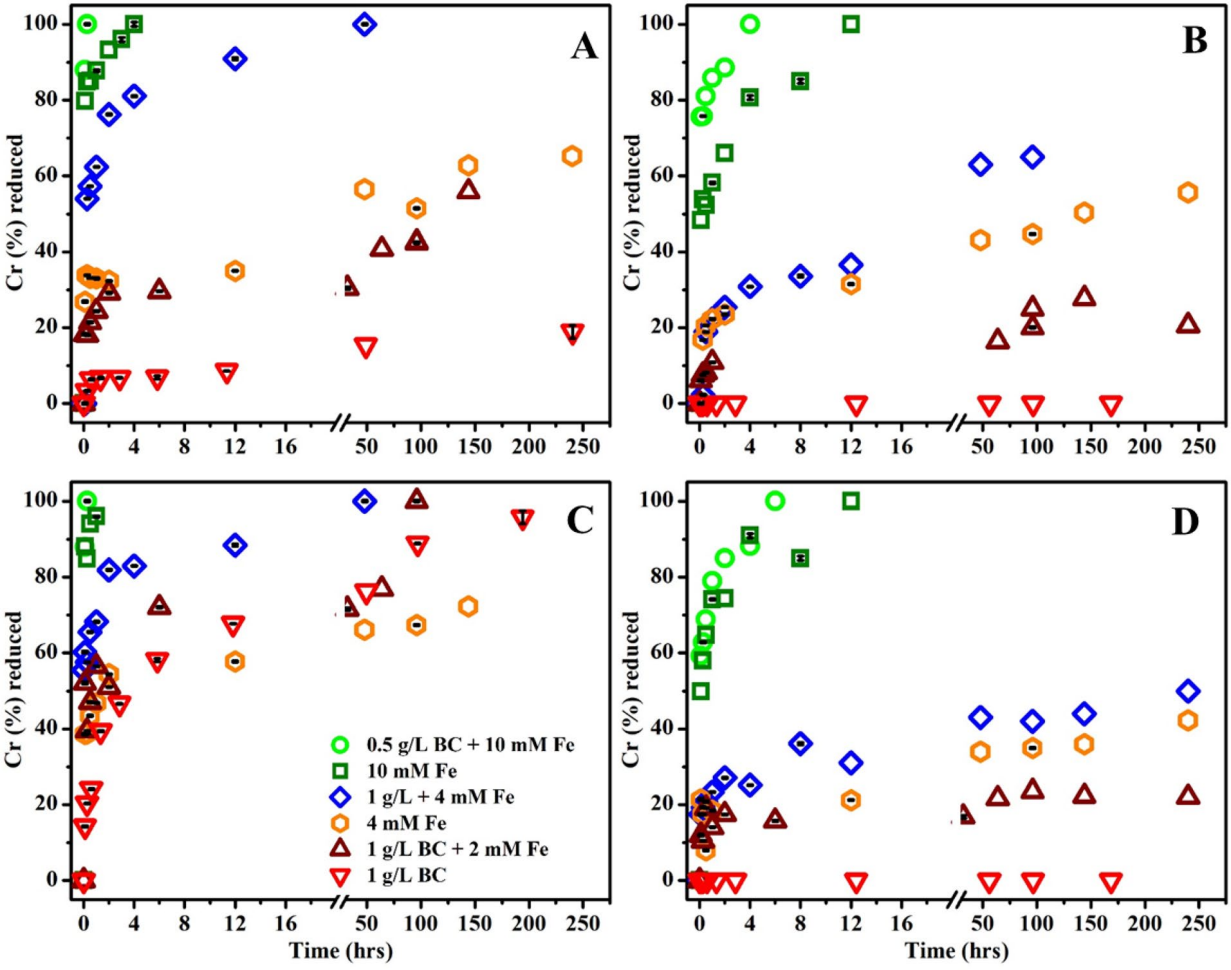
Cr(VI) reduction by MNP–BC; MNPs and BC at: (**A**) pH 2; (**B**) pH 3; (**C**) pH 5 and (**D**) pH 7.

The Cr(VI) reduction capacities of BC, MNPs and MNP–BC were also tested between pH 2–7 (Fig. 3). BC alone was effective in reducing Cr(VI) to Cr(III) at pH 2 (Fig. 3A), and after 10 days, 1 g L^−1^ BC was able to completely reduce a solution of 170 µM Cr(VI). At pH 3, only 20% of the total Cr(VI) in solution was reduced by BC in 10 days (Fig. 3B), and BC did not reduce a measureable concentration of Cr(VI) at pH 5 or 7, even at the lowest tested Cr(VI) concentration of 85 µM (Fig. 3C,D; Fig. S9). The reduction capacity of Cr(VI) to Cr(III) by BC is almost certainly controlled by the adsorption step. At a lower pH, where the BC surface is more positively charged, adsorption is more favorable and electron transfer from BC to Cr(VI) can occur, as shown in previous biochar studies^29,451^. The transformation products generated by pyrolysis of lignin and cellulose of the parent biomass are thought be the source of electron-donating moieties in biochar^51^. These moieties include phenolic and carboxylic functional groups to which Cr(VI) species can bind, while quinones and polycondensed aromatic structures (graphitic sheets) are a source of π-electrons and capable of transporting π-electrons to phenolic and carboxylic functional groups^21–22,51^. Our results demonstrate that Cr(VI) reduction by BC is influenced by protonation of the functional groups and Cr(VI) speciation.

The kinetics of the Cr(VI) reduction to Cr(III) by MNP–BC is much higher compared than those of MNPs at all pH values tested (pH 2–7) (Fig. 3). Cr(VI) reduction was measured concurrently with Cr(VI) sorption over time for each pH experiment, and we found that the Cr(VI) reduction kinetics closely matched Cr(VI) adsorption kinetics (Figs. 2 and 3). The results support our assertion that adsorption was the rate limiting step for Cr(VI) reduction by BC, MNPs and MNP–BC. The results from the reusability test showed that MNP–BC can be recycled multiple times to remove Cr(VI) from solution, as 10 mM Fe+ 1 gL^−1^ BC still can remove 80% of 170 µM of Cr(VI) from solution at its fifth reuse (Fig. S10). Since BC is conductive and enhances electron transfer along with preventing aggregation of MNPs, the cooperation between MNPs and BC leads to a higher efficiency in Cr(VI) adsorption and reduction by the composite across a wide range of pH. However, surface absorption of Cr(VI) should not impact the hydrodynamic size of the particles of magnetite in the composites. The strong cooperative behavior of MNP–BC for both adsorption and reductive immobilization make it a promising material for removal of Cr(VI) from aqueous solution.

### Effect of oxidants on reduction of Cr(VI)

In natural waters and wastewaters, oxidants can coexist with heavy metals^52–54^. Thus, it is essential to evaluate how oxidants could influence the reduction capacity of MNP–BC composites. The effects of dissolved oxygen (O_2_) and the most prevalent oxidants in drinking and wastewater systems [i.e., potassium permanganate (KMnO_4_), sodium chlorite (NaClO_2_) and sodium hypochlorite (NaClO)]^53^ on the reduction of Cr(VI) by MNP–BC were tested (Fig. S11). O_2_ did not have a measurable influence on the rate or extent of Cr(VI) reduction. However, the presence of KMnO_4,_ NaClO_2_ and NaClO slowed the kinetics of reduction. The removal efficiency of Cr(VI) was reduced from approximately 100–70% in the presence of KMnO_4_ and NaClO_2_, and reduced further to 40% by NaClO over the 10 day course of the experiments_._ The results clearly show that these oxidants impede Cr(VI) reduction by consuming electrons from MNP–BC. Nonetheless, the composites still removed Cr(VI) from solution, even in the presence of oxidants..

### Cr(VI) adsorption and reduction kinetics

The kinetics of adsorption and reduction are key factors in designing an adsorptive material for Cr(VI) removal from solution^34,44^. Adsorption proceeded rapidly for the first few hours of each experiment, and then gradually reached equilibrium (Fig. 3). Both pseudo-first-order and pseudo-second-order models yielded good fits of Cr(VI) adsorption to BC kinetics data (R^2^ *ca.* 0.9; Figs. S12A and S13A). In contrast, higher correlation coefficients were achieved using a pseudo-second-order model (R^2^ > 0.92) for Cr(VI) adsorption to MNP–BC than using a pseudo-first order model (R^2^ < 0.6), indicating that chemical adsorption could be the rate-limiting step in the reduction process for Cr(VI) by both BC and MNP–BC (Figs. S12B and S13B; Table S4). The adsorption processes may also be diffusion or intraparticle-diffusion controlled. Intraparticle diffusion reactions occur as soon as the reactants (i.e., sorbate) enter into solution^29,31–32,55^. BC is mesoporous as observed from the porosity analysis, and also hydrophobic. Thus, the process of wetting of biochar mesopores likely influences the Cr adsorption/reactions process in these mesopores. Therefore, the kinetics data were also analyzed using an intraparticle diffusion model to determine if the adsorption/reactions mechanisms by BC and MNP–BC were controlled by varying diffusion rates. The results indicate that the binding of Cr(VI) onto BC and MNP–BC is governed by both surface adsorption and intraparticle diffusion, followed by redox reactions that reduce Cr(VI) to Cr(III) (Figs. S12 and S13; Table S5).

The kinetics of Cr(VI) reduction by BC, MNPs and MNP–BC were additionally modeled to determine the order and rates of Cr(VI) reduction at varying pH. The reaction kinetics of a pseudo-first-order kinetic model are typically rate proportional to dissolved Cr(VI) concentration ([Cr(VI)_soln_])^56–60^ , according to:

1 $$ d\left[ {Cr\left( {VI} \right)_{sol} } \right]/dt = - K_{int} \left[ {Cr\left( {VI} \right)_{sol} } \right] $$

where, *K*_int_ (h^−1^) is the intrinsic first-order rate coefficient.

Previous studies determined that Cr(VI) reduction by biosorbents follows a pseudo-first-order model^29,59^. Our results show that a pseudo-first-order fits the experimental data of Cr(VI) reduction by BC at pH 2 fairly well (liner correlation coefficient R^2^ =  ~ 0.8), but provides a poor fit to reduction experiments conducted at pH 3 (R^2^ =  ~ 0.5)  (Fig. S14A,B). Since Cr(VI) reduction by BC was rapid during the first two hours, modeling the kinetics data using two pseudo-first-order kinetics relationships, one for the fast step (< 2 h) and a second for the slow step (> 2 h), provides the best fit to our experimental data (Fig. S14C,D). The reduction rate constant (*K*_*int*_) decreased with increasing pH and Cr(VI) concentration; a similar observation was also shown in other Cr(VI) reduction studies^57,58^ (Table S6). The higher Cr(VI) concentration could add more complexity to the system by changing chromate aqueous speciation and polymerization, thereby influencing the reduction rate^61^.

The reduction experiments by MNP–BC were modeled using second-order kinetics, according to the following rate law^57–61^

2 $$ \frac{{d\left[ {Cr\left( {VI} \right)} \right]}}{dt} = - K_{int} \left( {pH} \right)\left[ {Cr\left( {VI} \right)} \right]\left[ {Fe\left( {II} \right)} \right] $$

The second-order kinetics model provides better fit than does the pseudo-first-order model for MNPs and MNP–BC (Fig. S15). The Fe(II) concentration in the composite material also influenced the rate of Cr(VI) reduction by MNPs loading on MNP–BC. With decreasing Fe(II) concentration in the composite, the actual ate of chromate reduction (*K*obs) is decreased (Table S7). Overall, the kinetics models suggest that Cr(VI) reduction by BC followed first-order kinetics, and that the rate and order of the reduction depended on both pH and Cr concentration. Cr(VI) reduction could also be influenced by BC concentrations at acidic pH levels (e.g., pH 2 and 3). Since BC could not reduce measurable Cr(VI) at pH 5 and 7, it is expected that pH is the dominant factor for Cr(VI) reduction by BC. The 2nd order kinetics model best fits the results for Cr(VI) reduction by MNPs and MNP–BC, as Cr(VI) reduction was controlled by pH and Cr concentration as well as the Fe concentration in the MNPs and in MNP–BC.

### Cr(VI) adsorption and reduction mechanisms

To better understand the processes of adsorption and reduction, we studied the local molecular coordination environment of Cr on BC, MNPs and MNP–BC using XPS, FT-IR, XANES and extended X-ray adsorption fine structure (EXAFS) analyses. The XPS results indicate that after Cr(VI) adsorption, the intensity of the O 1s peak at the binding energy of 530.3 eV increased. This suggests that the Cr(VI) adsorption involved Fe–OH and C–OH functional groups, resulting in changes to the O1s intensity (Fig. S16). The FT-IR spectra show that the relative intensity of Fe–O peaks in MNPs, Fe–O and –OH peaks in MNP–BC, and C=O and –OH peaks in BC, shifted downward in the Cr-loaded samples (Fig. S17), suggesting that Cr(VI) complexed with these functional groups. Synchrotron based-XRF mapping of Cr(VI)-laden MNP–BC shows heterogeneous distribution of Cr on the composite surface (Fig. S18), while Fe had a varied correlation with Cr over the sample matrix (R^2^ = 0.66–1.00) suggesting that both Cr sorption and reduction might occur simultaneously for both MNPs and BC (Fig. S19).

**Figure 4:**
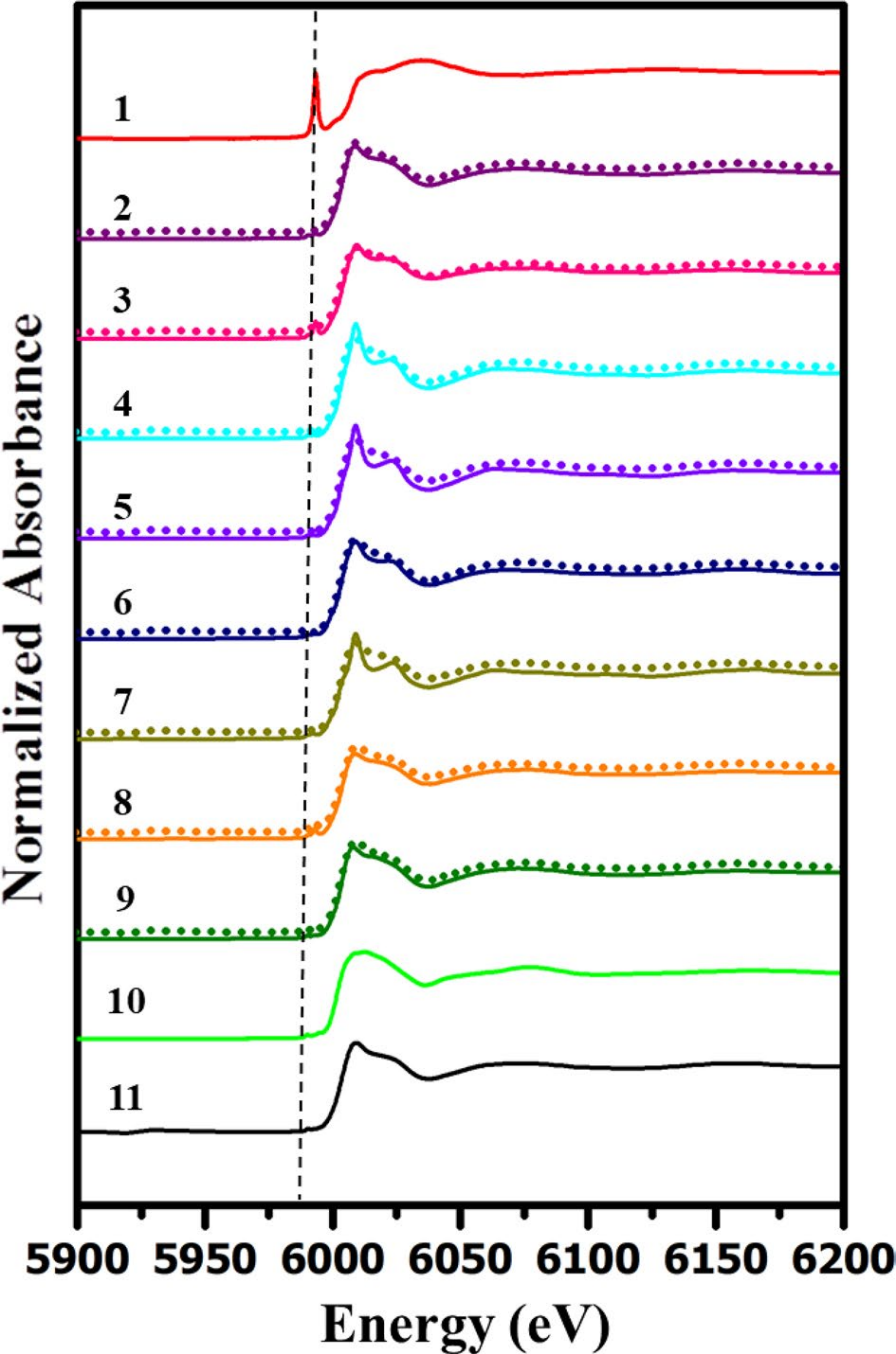
Cr K-edge XANES spectra of chromium references and Cr-loaded MNP–BC, MNPs and BC at different pH conditions (1) K_2_Cr_2_O_7_ standard; (2) 2 mM Fe+ 1 g L^−1^ BC at pH 7; (3) 4 mM Fe+ 1 g L^−1^ BC at pH 5; (4) 10 mM Fe+ 0.5 g L^−1^ BC at pH 5; (5) 10 mM MNP + 0.5 g L^−1^ BC at pH 7; (6) 10 mM MNP at pH 5; (**7**) 10 mM MNP at pH 7; (8) 1 g L^−1^ BC at pH 3; (9) 1 g L^−1^ BC at pH 2; (10) Cr(III) acetate standard and (11) Cr(OH)_3_ standard. Solid lines represent experimental data and dotted lines represent fit, respectively.

The XANES spectra of the Cr-laden samples clearly indicate the reduction of Cr(VI) to Cr(III) (Fig. 4). The Cr(VI) standard (K_2_Cr_2_O_7_) has a distinct and well-characterized pre-edge feature at 5990 eV, which is absent in the spectrum of trivalent Cr (Cr(OH)_3_). The linear combination fitting (LCF) of the XANES spectra show that more than 80% of the Cr(VI) reduced to Cr(III) by BC, MNPs and MNP–BC (Table S8).

**Figure 5:**
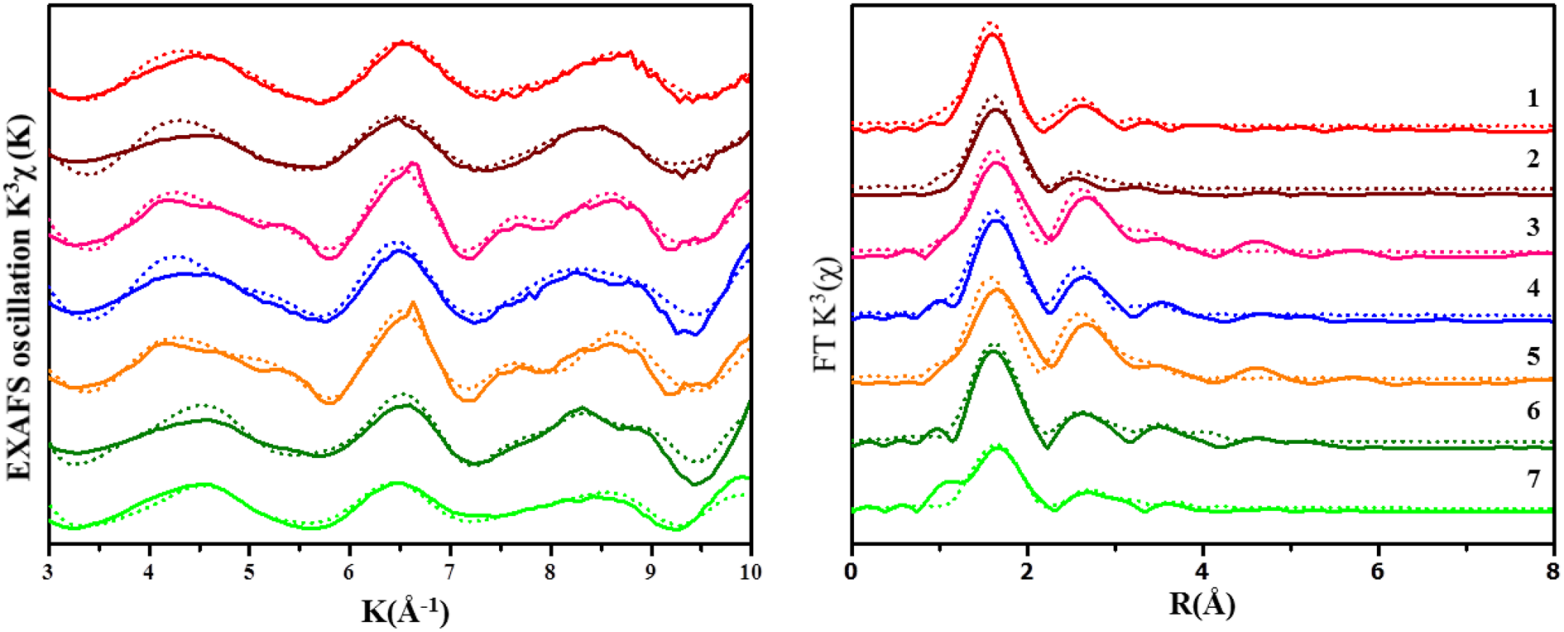
Cr K-edge EXAFS signals weighted by (**A**) k^3^ spectra and the (**B**) radial distribution function of Cr-loaded samples. (1) 2 mM Fe+ 1 g L^−1^ BC at pH 7; (2) 10 mM Fe+ 0.5 g L^−1^ BC at pH 5; (3) 10 mM MNP + 0.5 g L^−1^ BC at pH 7; (**4**) 10 mM MNP at pH 5; (5) 10 mM MNP at pH 7; (6) 1 g L^−1^ BC at pH 3 and (7) 1 g L^−1^ BC at pH 2. Solid lines represent experimental data and dotted lines represent fit, respectively.

The local coordination environment of Cr associated with BC, MNPs and MNP–BC was analyzed using EXAFS fitting (Fig. 5). Converted to R space, the first shell at 1.98–2.02 Å in the samples are consistent with literature values Cr(III)–O bonds having lengths of 1.98 Å (Table S9). The coordination number of Cr(III)–O is 6 in the samples, likely representing an octahedral geometry. Since BC contains a variety of ligands, including carboxylate, quinone and phenolic groups, Cr(III) can complex with any of them^34^. The second shell present in all samples is consistent with Cr–Cr bonding at a distance ~ 3.1 Å, and indicates the presence of amorphous Cr(OH)_3_ precipitates, formed as a result of Cr(VI) reduction to Cr(III). The Cr 2p at binding energy of 577.20 eV from XPS analysis also supports the presence of a Cr(OH)_3_ precipitate (Fig. S20)^62^. The Cr-C shells in BC and MNP–BC are indicative of inner-sphere surface complexation. Cr–Fe pathways in MNPs and MNP–BC suggest that chromite nanoparticles (Cr_2_FeO_4_) formed on the surface of MNP and MNP–BC. To test this hypothesis, XANES modeling was carried out, and the results showed that of Cr local structural environment containing Cr_2_FeO_4_ nanoparticles exists in the sample system (Fig. S21). The size of the Cr_2_FeO_4_ particle in the sample system is between approximately 0.5 µm to less than 1.0 nm in diameter.

### Crystal transformation of magnetite nanoparticles

The binding of MNPs on BC was analyzed by FT-IR (Fig. S17). The free stretching C=O band in BC is usually assigned at 1590 cm^−1^. In the composite upon complexation with MNPs, the intensity of the C=O_BC_ band in MNP–BC is decreased. However, the interaction between BC and MNPs does not appear to be affected by Cr(VI) adsorption, as shown by the unchanging C=O band, indicative of strong binding and complexation with magnetite^34–35^. The DOC leaching data shows that less than 2% of the DOC leached from BC and MNP–BC composites, indicating that BC is stable at low pH.

To better understand the transformation of magnetite in the composites after Cr(VI) adsorption and reduction, Cr(VI)-loaded MNPs and MNP–BC samples were analyzed with XRD, XPS, Fe K-edge XANES and EXAFS, and ^57^Fe Mössbauer spectroscopy. The XRD and XPS spectra indicated no detectable difference between magnetite in the original MNP–BC at higher Fe concentration (10 mM Fe+ 1 g L^−1^ BC) and Cr(VI)-loaded MNP–BC implying no transformation from magnetite to maghemite (Figs. S22 and S23). However, XRD analysis of Cr(VI)-loaded MNP–BC at the lower Fe concentration (2 mM Fe+ 1 g L^−1^ BC) showed that the diffraction peaks at 2θ = 35.42° and 43.05°, which correspond to the (311) and (400) planes, respectively, are not well indexed to the face-centered cubic inverse spinal structure of crystalline Fe_3_O_4_, indicating some transformation in magnetite (S22C and S22D).

Fe 2p_1/2_ and Fe K-edge XANES of Cr(VI)-loaded MNP–BC (Figs. S23 and S24) showed some transformation of magnetite in MNP–BC after Cr(VI) reduction to Cr(III). The coordination number and the inter atomic distance calculated from Fe K-edge EXAFS spectra (*k*^3^*·χ*(*k*)) fitting shows that the samples exhibited better fits to the magnetite crystallography (Fig. S25 and Table S10) as compared to oxidized Fe mineral phases, suggesting that magnetite did not convert completely to an oxidized Fe(III) mineral. The linear combination fitting (LCF) (Table S11) of the Fe XANES spectra showed that magnetite had partially transformed to oxidized Fe phases (likely ferrihydrite and goethite) after complete reduction of 350 µM Cr(VI) to Cr(III) in 4 days, implying that the reduction of Cr(VI) to Cr(III) consumed a fraction of the Fe(II) (Fig. S24).

**Figure 6:**
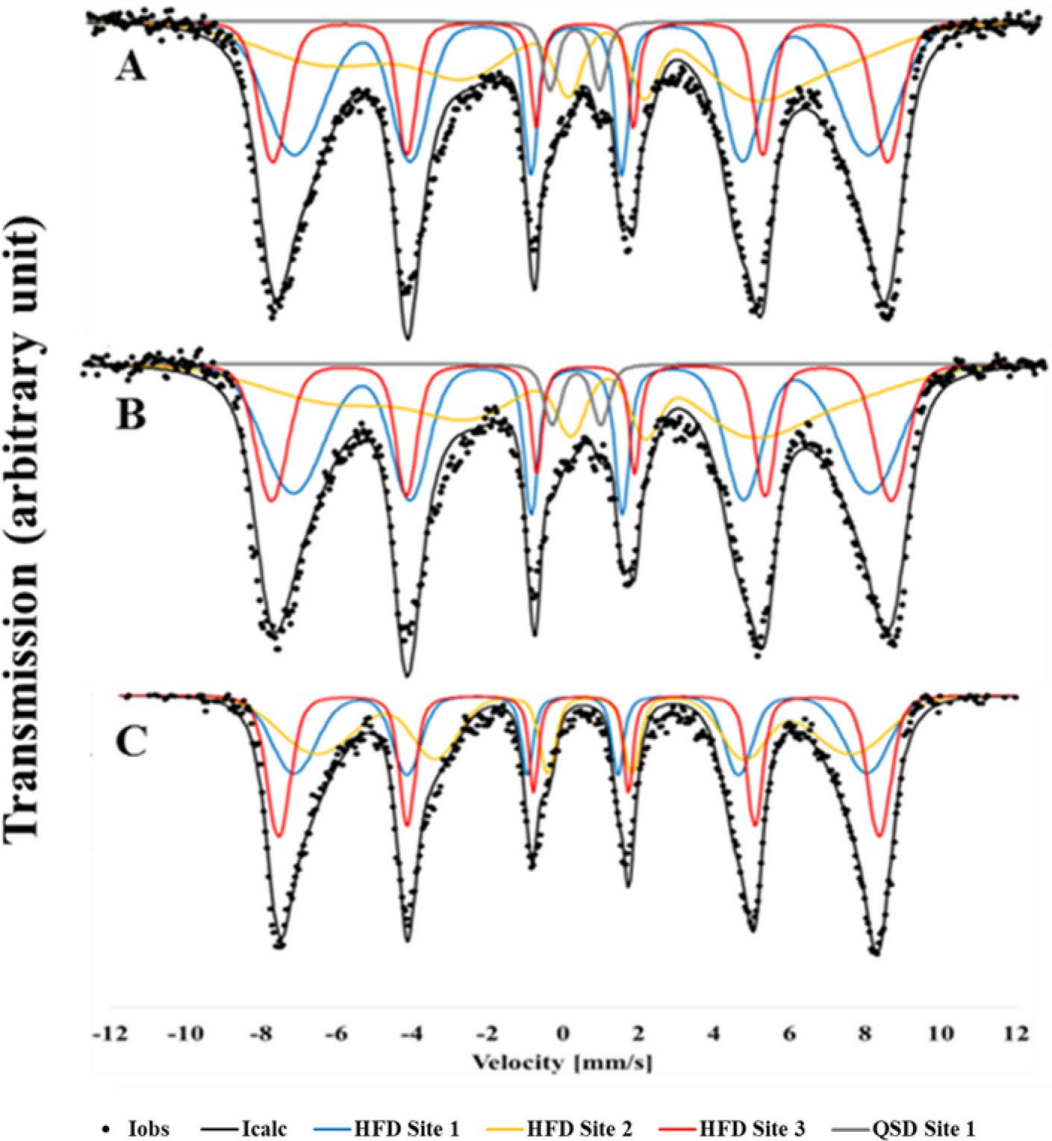
Mössbauer spectroscopy of Cr-loaded MNP–BC (10 mM Fe+ 1 g L^−1^ BC + 350 µM Cr(VI)) at different reaction time with Cr(VI). (**A**) t = 0; (**B**) t = 2 days and (**C**) t = 4 days at pH 7. Raw data (black dots); Sum of all fits (black); HFD Site 1—Oh = octahedral coordinated magnetite sextets (blue); HFD Site 2—superparamagnetic phase (yellow); HFD Site 3—Td = tetrahedral coordinated magnetite sextets (red); QSD Site 1—Fe(III) phase (grey).

Cr-loaded MNP–BC was further analyzed by Mössbauer spectroscopy. The spectra of Cr-loaded MNP–BC (10 mM Fe+ 1 g L^−1^ BC) are dominated by two magnetically ordered hyperfine sextets (HFD Site 1 and HFD Site 3; Table S12) which constitute approximately 65% of the complete spectral area. The hyperfine parameters revealed these sextets to correspond to octahedral and tetrahedral lattice sites, respectively (Fig. 6). However, accurate fitting of the spectra also required the inclusion of a collapsed sextet in addition to a doublet which are indicative of a superparamagnetic, or poorly ordered phase. This collapsed sextet was present in all samples, but the doublet was only present for samples collected at t = 0 and t = 2 days. It was not possible to identify either of these components, however they likely correspond to superparamagnetic magnetite, or potentially a maghemite like phase meaning that it is not possible to explicitly exclude the presence of maghemite in these samples. The prominent doublet at lower Fe concentration (2 mM Fe+ 1 g L^−1^ BC) in MNP–BC, located in the middle of both spectra, accounts for about 10% of the spectral area and likely corresponds to a paramagnetic Fe(III) phase, such as lepidocrocite or nano-goethite (Fig. S26).

## Conclusions

The reductive immobilization of Cr(VI) to nontoxic Cr(III) via a coupled reduction–sorption process is amongst the best remediation strategies to treat Cr(VI)-contaminated water. Magnetite and biochar can effectively remove Cr(VI) from aqueous solutions at low pH, but their individual efficiencies decrease at neutral pH^13^. Here we demonstrate that magnetite nanoparticle–biochar composites provide a robust, reusable adsorbent that can transform Cr(VI) to Cr(III). The adsorption–reduction processes suggest that the strong cooperation between MNP and BC underpins the improved adsorption and reduction capacity. Moreover, the kinetics of Cr(VI) adsorption by MNP–BC is rapid, reaching equilibrium within an hour. The chemical process of Cr(VI) removal by MNP–BC occurs through adsorption and intraparticle diffusion, followed by redox reactions that reduce chromium to its trivalent state. Spectroscopic analyses showed the complete reduction Cr(VI) to Cr(III) by MNP–BC, leading to an immobilized Cr(III)-complex including Cr(OH)_3_ and chromite (Cr_2_FeO_4_). The ability of the MNP–BC composite to both absorb and reduce redox-sensitive metals suggests it as a cost-effective, promising and reliable sorbent in the treatment of contaminated water.

## materials and methods

### Preparation of Fe_3_O_4_ nanoparticles (MNPs) and Fe_3_O_4_–biochar composites (MNP–BC)

The MNPs and MNP–BC composites were prepared according to Liu et al*.*^39^ and Yuan et al*.*^40^. Briefly, 2 g FeCl_3_·6H_2_O and 1 g FeCl_2_·4H_2_O were dissolved in 200 mL water that had been bubbled with N_2_ gas for 30 min. While continuing to bubble with N_2_, the solution was vigorously stirred for 5 min at 60 °C. A solution of 1.5 M NH_4_OH was added dropwise into solution until the pH value of the mixture reached 8.0 and a black precipitate formed. The mixture was then aged for 30 min at 60 °C. The solid precipitate was centrifuged and separated from water, washed four times with water and then dried at 40 °C in a vacuum oven. To prepare MNP–BC, BC was added to the initial ferric/ferrous chloride solution described above, at several MNP to BC ratios. The addition of NH_4_OH and subsequent preparation steps were identical to those conducted for the synthesis of MNPs.

### Characterization of adsorbents

The BC, MNPs and MNP–BC were characterized by Co K-alpha X-ray powder diffraction (XRD, Rikagu Ultima IV), high resolution transmission electron microscopy equipped with selected area diffraction (HRTEM-SAED, JEM-ARM200cF S/TEM) and scanning electron microscopy (SEM, Zeiss EVO® MA 15). Fourier transform infrared spectroscopy (FTIR, Thermo Nicolet 8700) was employed to analyze the molecular structure. The particle size distributions and zeta potential of BC, MNPs and MNP–BC were measured using dynamic light scattering (DLS) and electrophoretic light scattering (ELS) analyses, respectively (ZetaSizer Nano, Malvern Instruments, UK). X-ray photoelectron spectroscopy (XPS) measurements were carried out using a Kratos Axis 165 instrument to determine the functional groups and Cr and Fe speciation. The detailed procedure of these methods is given in the Supporting Information (SI) section. Potentiometric titrations were carried out to determine the protonation constants (K_a_) and corresponding site concentrations of proton-active surface functional groups, as described in Alam et al*.*^7,20^ To determine the sheet resistance of BC, it was mixed with a polyethylene oxide (PO) polymer. The sheet resistance of the BC and PO composites at different weight ratios (20 mg:200 mg; 100 mg:100 mg; 200 mg:20 mg) was measured at room temperature using a linear four points probe with 1 mm spacing (Lucas Pro-4 4000, USA) and a Keithley 2601A SourceMeter. Dry combusted and water extracted total organic carbon (TOC) of BC were determined using a TOC analyzer (Shimadzu TOC-V/TN). To ascertain the ratios of Fe(II) and Fe(III) in MNPs, the ferrozine method^43^ was used.

### Cr(VI) adsorption and reduction experiments

Batch adsorption and reduction kinetics experiments were conducted at various initial Cr(VI) concentrations (85 µM, 170 µM and 350 µM) and sorbent concentrations (1 g L^−1^ BC, 10 mM Fe, 4 mM Fe, 0.5 g L^−1^ BC + 10 mM Fe, 1 g L^−1^ BC + 4 mM Fe, and 1 g L^−1^ BC + 2 mM Fe) at pH 2, 3, 5 and 7. Initially, a 10 g L^−1^ MNPs and MNP–BC stock slurry was prepared. The reported Fe and BC + Fe iron concentrations were calculated from dilutions of the stock MNPs and addition of MNP to the MNP–BC slurry, respectively. The release of Fe from BC was negligible (less the few ppb) at the tested pH values. The adsorption and reduction kinetics experiments were run in duplicate and the standard deviations were less than 5%. Details of the Cr (VI) adsorption and reduction experiments and kinetics modeling are given in the SI section.

### Solid phase analyses after adsorption and reduction

Synchrotron X-ray absorption spectroscopy (XAS) analyses were carried out at the Hard X-ray Microanalysis beamline (HXMA 06ID-1) of the Canadian Light Source (CLS). Synchrotron-based X-ray fluorescence (XRF) mapping was carried out on thin sections of Cr-loaded BC, MNPs and MNP–BC at the Very Sensitive Elemental and Structural Probe Employing Radiation beamline (VESPERS 07B2-1) at CLS. ^57^Fe Mössbauer spectroscopy was conducted to characterize changes to Fe bearing mineral phases in MNP–BC exposed to different concentrations of Cr(VI). The detailed XAS, synchrotron-XRF and ^57^Fe Mössbauer spectroscopy method are described in the SI. Cr-loaded BC, MNPs and MNP–BC samples were further analyzed by XRD, FTIR and XPS to determine the crystallographic transformation of MNPs and the functional groups that interacted with Cr after the adsorption and reduction processes.

## Supplementary information

Supplementary Information 1.

Supplementary Information 2.

## References

[1] Fendorf S, Wielinga BW, Hansel CM (2002). Chromium transformations in natural environments: the role of biological and abiological processes in chromium(VI) reduction. Int. Geol. Rev..

[2] Eary LE, Rai D (1988). Chromate removal from aqueous wastes by reduction with ferrous ion. Environ. Sci. Technol..

[3] Patterson RR, Fendorf S, Fendorf M (1997). Reduction of hexavalent chromium by amorphous iron sulfide. Environ. Sci. Technol..

[4] Jung Y, Choi J, Lee W (2007). Spectroscopic investigation of magnetite surface for the reduction of hexavalent chromium. Chemosphere.

[5] He YT, Traina SJ (2005). Cr(VI) reduction and immobilization by magnetite under alkaline pH conditions: the role of passivation. Environ. Sci. Technol..

[6] Sass BM, Rai D (1987). Solubility of amorphous chromium(III)–iron(III) hydroxide solid solutions. Inorg. Chem..

[7] Rai D, Sass BM, Moore DA (1987). Chromium(III) hydrolysis constants and solubility of chromium(III) hydroxide. Inorg. Chem..

[8] Chowdhury SR, Yanful EK (2010). Arsenic and chromium removal by mixed magnetite–maghemite nanoparticles and the effect of phosphate on removal. J. Environ. Manag..

[9] He YT, Chen C, Traina SJ (2004). Inhibited Cr(VI) reduction by aqueous Fe(II) under hyperalkaline conditions. Environ. Sci. Technol..

[10] Rebodos RL, Vikesland PJ (2010). Effects of oxidation on the magnetization of nanoparticulate magnetite. Langmuir.

[11] Demangeat E, Pedrot M, Dia A, Bouhnik-le-Coz M, Grasset F, Hanna K, Kamagate M, Cabello-Hurtado F (2018). Colloidal and chemical stabilities of iron oxide nanoparticles in aqueous solutions: the interplay of structural, chemical and environmental drivers. Environ. Sci. Nano.

[12] Campos AFC, de Oliveira HAL, da Silva FN, da Silva FG, Coppola P, Aquino R, Mezzi A, Depeyrot J (2019). Core–Shell bimagnetic nanoadsorbents for hexavalent chromium removal from aqueous solutions. J. Hazard Mater..

[13] Peterson ML, White AF, Brown GE, Parks GA (1997). Surface passivation of magnetite by reaction with aqueous Cr(VI): XAFS and TEM results. Environ. Sci. Technol..

[14] Georgakilas V, Perman JA, Tucek J, Zboril R (2015). Broad family of carbon nanoallotropes: classification, chemistry, and applications of fullerenes, carbon dots, nanotubes, graphene, nanodiamonds, and combined superstructures. Chem. Rev..

[15] Yao Y, Gao B, Wu F, Zhang C, Yang L (2015). Engineered biochar from biofuel residue: characterization and its silver removal potential. ACS Appl. Mater. Interfaces.

[16] Cao XD, Ma LN, Gao B, Harris W (2009). Dairy-manure derived biochar effectively sorbs lead and atrazine. Environ. Sci. Technol..

[17] Chen Z, Chen B, Chiou CT (2012). Fast and slow rates of naphthalene sorption to biochars produced at different temperatures. Environ. Sci. Technol..

[18] Thompson KA, Shimabuku KK, Kearns JP, Knappe DRU, Summer S, Cook SM (2016). Environmental comparison of biochar and activated carbon for tertiary wastewater treatment. Environ. Sci. Technol..

[19] Alam MS, Cossio M, Robinson L, Kenney JPL, Wang X, Konhauser KO, MacKenzie MD, Ok YS, Alessi DS (2016). Removal of organic acids from water using biochar and petroleum coke. Environ. Technol. Innov..

[20] Alam MS, Swaren L, Gunten KV, Cossio M, Robbins LJ, Flynn SL, Konhauser KO, Alessi DS (2018). Application of surface complexation modeling to trace metals uptake by biochar-amended agricultural soils. Appl. Geochem..

[21] Yu L, Yuan Y, Tang J, Wang Y, Zhou S (2015). Biochar as an electron shuttle for reductive dechlorination of pentachlorophenol by Geobacter sulfurreducens. Sci. Rep..

[22] Keiluweit M, Nico PS, Johnson MG, Kleber M (2010). Dynamic molecular structure of plant biomass-derived black carbon (biochar). Environ. Sci. Technol..

[23] Kappler A, Wuestner ML, Ruecker A, Harter J, Halama M, Behrens S (2014). Biochar asan electron shuttle between bacteria and Fe(III) minerals. Environ. Sci. Technol. Lett..

[24] Xu S, Adhikari D, Huang R, Zhang H, Tang Y, Roden E, Yang Y (2016). Biochar facilitated microbial reduction of hematite. Environ. Sci. Technol..

[25] Xu J, Yin Y, Tan Z, Wang B, Guo X, Li X, Liu J (2019). Enhanced removal of Cr(VI) by biochar with Fe as electron shuttles. J. Environ. Sci..

[26] Xu X, Huang H, Zhang Y, Xu Z, Cao X (2019). Biochar as both electron donor and electron shuttle for the reduction transformation of Cr(VI) during its sorption. Environ. Pol..

[27] Wan Z, Cho D-W, Tsang DCW, Li M, Sun T, Verpoort F (2019). Concurrent adsorption and micro-electrolysis of Cr(VI) by nanoscale zerovalent iron/biochar/Ca-alginate composite. Environ. Pol..

[28] Rajapaksha AU, Alam MS, Chen N, Alessi DS, Igalavithana AD, Tsang DCW, Ok YS (2018). Removal of hexavalent chromium in aqueous solutions using biochar: chemical and spectroscopic investigations. Sci. Total Environ..

[29] Cossio, M. *Mechanisms of the Reductive Immobilization of Hexavalent Chromium by Wheat Straw Biochar*. M.Sc. Thesis. University of Alberta (2017).

[30] Beller HR, Yang L, Varadharajan C, Han R, Lim HC, Karaoz U, Molins S, Marcus MA, Brodie EL, Steefel CI (2014). Divergent aquifer biogeochemical systems converge on similar and unexpected Cr(VI) reduction products. Environ. Sci. Technol..

[31] Cheng Y, Yan F, Huang F, Chu W, Pan D, Chen Z, Zheng J, Yu M, Lin Z, Wu Z (2010). Bioremediation of Cr(VI) and immobilization as Cr(III) by *Ochrobactrum anthropi*. Environ. Sci. Technol..

[32] Deshpande K, Cheung S, Rao MS, Dave BC (2005). (2005) Efficient sequestration and reduction of hexavalent chromium with organosilica sol–gels. J. Mater. Chem..

[33] Zaitseva N, Zaitsev V, Walcarius A (2013). Chromium (VI) removal via reduction–sorption on bi-functional silica adsorbents. J. Hazard. Mater..

[34] Jiang W, Cai Q, Xu W, Yang M, Cai Y, Dionysiou DD, O’Shea KE (2014). Cr(VI) adsorption and reduction by humic acid coated on magnetite. Environ. Sci. Technol..

[35] Wang T, Zhang L, Li C, Yang W, Song T, Tang C, Meng Y, Dai S, Wang H, Chai L, Luo J (2015). Synthesis of core shell magnetic Fe_3_O_4_@poly(m-phenylenediamine) particles for chromium reduction and adsorption. Environ. Sci. Technol..

[36] Mandal S, Sarkar B, Bolan N, Ok YS, Naidu R (2017). Enhancement of chromate reduction in soils by surface modified biochar. J. Environ. Manag..

[37] Su H, Fang Z, Tsang PE, Zheng L, Cheng W, Fang J, Zhao D (2016). Remediation of hexavalent chromium contaminated soil by biochar-supported zero-valent iron nanoparticles. J. Hazard. Mater..

[38] Hu Q, Zhu Y, Hu B, Lu S, Sheng G (2018). Mechanistic insights into sequestration of U(VI) toward magnetic biochar: batch, XPS and EXAFS techniques. J. Environ. Sci..

[39] Liu JF, Zhao ZS, Jiang GB (2008). Coating Fe_3_O_4_ magnetic nanoparticles with humic acid for high efficient removal of heavy metals in water. Environ. Sci. Technol..

[40] Yuan P, Fan M, Yang D, He H, Liu D, Yuan A, Zhu J, Chen T (2009). Montmorillonite supported magnetite nanoparticles for the removal of hexavalent chromium [Cr (VI)] from aqueous solutions. J. Hazard. Mater..

[41] Ezzaier H, Marins JA, Claudet C, Hemery G, Sandre O, Kuzhir O (2018). Kinetics of aggregation and magnetic separation of multicore iron oxide nanoparticles: effect of the grafted layer thickness. Nanomaterials.

[42] Antone AJ, Sun Z, Bao Y (2019). Preparation and application of iron oxide nanoclusters. Magnetochemistry.

[43] Viollier E, Inglett PW, Hunter K, Roychoudhury AN, Van Cappellen P (2000). The ferrozine method revisted: Fe(II)/Fe(III) determination in natural waters. Appl. Geochem..

[44] Venkateswarlu S, Lee D, Yoon M (2016). Bioinspired 2D-carbon flakes and Fe_3_O_4_ nanoparticles composite for arsenite removal. ACS Appl. Mater. Interfaces.

[45] Chen Z, Xiao X, Chen B, Zhu L (2015). Quantification of chemical states, dissociation constants and contents of oxygen-containing groups on the surface of biochars produced at different temperatures. Environ. Sci. Technol..

[46] Cuenca JA, Bugler K, Taylor S, Morgan D, Williams P, Bauer J, Porch A (2016). Study of the magnetite to maghemite transition using microwave permittivity and permeability measurements. J. Phys. Condens. Matter.

[47] Herbelin, A. & Westall, J. *FITEQL-a Computer Program for Determination of Chemical Equilibrium Constants from Experimental Data Version 3.2 User's Manual*. Department of Chemistry, Oregon State University, Corvallis, OR, Report.: 1996, 96–01 (1996).

[48] Chia CH, Gong B, Joseph SD, Marjo CE, Munroe P, Rich AM (2012). Imaging of mineral-enriched biochar by FTIR, Raman and SEM–EDX. Vib. Spectrosc..

[49] Chen B, Zhou D, Zhu L (2008). Transitional adsorption and partition of nonpolar and polar aromatic contaminants by biochars of pine needles with different pyrolytic temperatures. Environ. Sci. Technol..

[50] Li M, Liu Q, Lou Z, Wang Y (2014). Method to characterize acid-base behavior of biochar: site modeling and theoretical simulation. ACS Sustain. Chem. Eng..

[51] Yuan Y, Bolan N, Prévoteau A, Vithanage M, Biswas JK, Ok YS, Wang H (2017). Applications of biochar in redox-mediated reactions. Bioresour. Technol..

[52] Rogers, N. D. *Chromium Oxidation by Disinfectants and Oxidants Used in Drinking Water Treatment*. All Graduate Theses and Dissertations*.* Paper 5028. Utha State University (2016).

[53] Schroeder DC, Lee GF (1975). Potential transformations of chromium in natural waters. Water,Air Soil Pollut..

[54] Sorlini S, Gialdini F, Biasibetti M, Collivignarelli C (2014). Influence of drinking water treatments on chlorine dioxide consumption and chlorite/chlorate formation. Water Res..

[55] Tran HN, You SJ, Hosseini-Bandegharaei A, Chao HP (2017). Mistakes and inconsistencies regarding adsorption of contaminants from aqueous solutions: a critical review. Water Res..

[56] Eary LE, Rai D (1987). Kinetics of chromium(III) oxidation to chromium(VI) by reaction with manganese dioxide. Environ. Sci. Technol..

[57] Buerge IJ, Hug SJ (1997). Kinetics and pH dependence of chromium(VI) reduction by iron(II). Environ. Sci. Technol..

[58] Alowitz MJ, Scherer MM (2002). Kinetics of nitrate, nitrite, and Cr(VI) reduction by iron metal. Environ. Sci. Technol..

[59] Hsu NH, Wang SL, Lin YC, Sheng GD, Lee JF (2009). Reduction of Cr(VI) by crop-residue-derived black carbon. Environ. Sci. Technol..

[60] Hu X, Wang J, Liu Y, Li X, Zeng G, Bao Z, Zeng X, Chen A, Long F (2011). Adsorption of chromium (VI) by ethylenediamine-modified cross-linked magnetic chitosan resin: isotherms, kinetics and thermodynamics. J. Hazard. Mater..

[61] Park D, Yun YS, Ahn CK, Park JM (2007). Reduction of Hexavalent chromium with the brown seaweed Ecklonia biomass. Environ. Sci. Technol..

[62] Chowdhury SR, Yanful EK, Pratt AR (2012). Chemical states in XPS and Raman analysis during removal of Cr(VI) from contaminated water by mixed maghemite–magnetite nanoparticles. J. Hazard. Mater..

