# Supplementary material for: Reusable magnetite nanoparticles–biochar composites for the efficient removal of chromate from water
Source: Sci Rep. 2020 Nov 4;10:19007. doi: 10.1038/s41598-020-75924-7 (PMC7642354; doi:10.1038/s41598-020-75924-7)
Supplement: Supplementary file 1 — Supplementary Information 1. [file 41598_2020_75924_MOESM1_ESM.docx]

**Supporting Information**

Detailed discussion for materials, biochar characterization, Cr(VI) adsorption and reduction experiments, adsorption modeling, solid phase analyses after adsorption and reduction and Mössbauer spectrometry analysis. Sheet resistance (Figure S1), Size distribution of MNP (Figure S2), SEM images (Figure S3), Spectroscopic analyses (Figure S4), XRD patterns (Figure S5), Potentiometric titration (Figure S6), Speciation diagram of Cr(VI) and Cr(III) (Figure S7), Zeta potentials (Figure S8), Cr(VI) reduction by 1gL^-1^ BC at different pH conditions (Figure S9), Reusability of the MNP-BC composites (Figure S10), Effects of dissolved oxygen (DO) and selected oxidants on Cr(VI) reduction (Figure S11), Adsorption kinetics and intraparticle diffusion for Cr(VI) adsorption and reduction on BC and MNP-BC at different conditions (Figure S12 to S15), XPS survey O 1s spectra after Cr(VI) adsorption and reduction (Figure S16), FT-IR spectra after Cr(VI) adsorption and reduction (Figure S17), XRF map of Cr distribution (Figure S18), Correlation matrix of Fe and Cr (Figure S19), Cr 2p XPS spectra of Cr(VI) loaded on BC and MNP-BC (Figure S20), XANES modeled Cr_2_FeO_4_ structure (Figure S21), XRD patterns of MNPs and MNP-BC over time with or without Cr sorption (Figure S22), Fe 2p XPS spectra of MNPs and MNP-BC with or without Cr sorption (Figure S23), Fe K-edge XANES spectra (Figure S24), Fe K-edge EXAFS signals (Figure S25) and Mössbauer spectroscopy (Figure S26). Supplemental tables include elemental composition of BC (Table S2), pKas and site concentrations (Table S3), Kinetics rate constants for adsorption, reduction and intraparticle diffusion of Cr(VI) (Table S4 to S7), LCF results Cr K-edge XANES (Table S8), R-space curve fitting results of Cr K-edge EXAFS data (Table S9), LCF results Fe K-edge XANES (Table S10), R-space curve fitting results of Fe K-edge EXAFS data (Table S11) and Fitting results of Mössbauer spectroscopy (Table S12).
