# Supplementary material for: Reusable magnetite nanoparticles–biochar composites for the efficient removal of chromate from water
Source: Sci Rep. 2020 Nov 4;10:19007. doi: 10.1038/s41598-020-75924-7 (PMC7642354; doi:10.1038/s41598-020-75924-7)
Supplement: Supplementary file 2 — Supplementary Information 2. [file 41598_2020_75924_MOESM2_ESM.docx]

**Supporting Information**

**Reusable Magnetite Nanoparticles – Biochar Composites for the Efficient Removal of Chromate from Water**

Md. Samrat Alam^1*‖^, Brendan Bishop^1^, Ning Chen^2^, Salman Safari^1^, Viola Warter^3^, James M. Byrne^3^, Tyler Warchola^1^, Andreas Kappler^3^, Kurt O. Konhauser^1^ & Daniel S. Alessi^1*^

^1^Department of Earth & Atmospheric Sciences, 1-26 Earth Sciences Building, University of Alberta, Alberta, T6G 2E3, Canada

^2^Canadian Light Source Inc., University of Saskatchewan, 114 Science Place, Saskatoon, SK, S7N 0X4, Canada

^3^Geomicrobiology, Center for Applied Geoscience, University of Tübingen, 72076 Tübingen, Germany

*Corresponding Author. Phone: +1-587-783-9848; Email: [samrat.alam@utoronto.ca](mailto:samrat.alam@utoronto.ca); alessi@ualberta.ca

^‖^Current address: Department of Earth Sciences, University of Toronto, 22 Russell Street, Toronto, ON, M5S 3B1, Canada

Supporting Information consists of 36 pages, including 26 figures and 12 tables

## List of Supplemental Figures

## Figure S1: Sheet resistance of BC at different concentration.

## Figure S2: Size distribution of MNPs at different pH conditions.

Figure S3. SEM images of (A) BC; (B) MNPs; (C) MNP-BC (high Fe concentration); (D) back scattered electron (BSE) map of BC in thin section; (E) BSE map of MNP-BC in thin section; and (F) EDS of MNP-BC.

Figure S4. Spectroscopic analyses of MNPs, BC, and MNP-BC. (A) FT-IR spectra of MNPs, MNP-BC and BC; (B) Zeta potentials of MNPs, MNP-BC and BC; XPS spectra (C-F) (C) Fe 2p of MNPs; (D) Fe 2p of MNP-BC; (E) C 1s of MNP-BC and (F) C 1s of BC.

Figure S5: XRD patterns of (A) MNPs and (B) MNP-BC.

Figure S6: Potentiometric titration of (A) BC; (B) MNPs and (C) MNP-BC.

Figure S7: Speciation diagram of Cr(VI) and Cr(III). (A) 350 µM Cr(VI); (B) 170 µM Cr(VI); (C) 85 µM Cr(VI) and (D) Cr(III).

Figure S8: Zeta potentials of MNPs, MNP-BC and BC with and without Cr-sorption.

Figure S9: Cr(VI) reduction by 1gL^-1^ BC at different pH conditions. (A) 350 µM Cr(VI); (B) 170 µM Cr(VI); and (C) 85 µM Cr(VI).

Figure S10: Reusability of the MNP-BC composites.

Figure S11. Effects of dissolved oxygen (DO) and selected oxidants on Cr(VI) reduction by MNP-BC. (A) 10 mM Fe + 1 gL^-1^ BC (no oxidants); (B) DO; (C) KMnO_4_; (D) NaClO_2_ and (E) NaClO.

Figure 12. Adsorption kinetics and intraparticle diffusion for Cr(VI) adsorption on BC and MNP-BC. (A) Cr(VI) adsorption on BC fitted with pseudo-first-order; (B) Cr(VI) adsorption on MNP-BC fitted with pseudo-second-order models and (C,D) intraparticle diffusion plot for Cr(VI) adsorption on BC and MNP-BC, respectively.

Figure S13. Adsorption kinetics for Cr(VI) adsorption on BC and MNP-BC. (A) Cr(VI) adsorption on BC fitted with pseudo-second-order, and (B) Cr(VI) adsorption on MNP-BC fitted with pseudo-first-order models.

Figure S14. Reduction kinetics for Cr(VI) reduction by BC at different Cr concentrations and pH conditions. (A,B) Cr(VI) reduction on BC fitted with pseudo-first-order model at pH 2 and 3, respectively, and (C,D) Cr(VI) reduction on BC fitted with pseudo-first-order model using fast and slow kinetics at pH 2 and 3, respectively.

Figure S15. Reduction kinetics for Cr(VI) reduction by MNP-BC and MNPs at different concentrations and pH conditions. (A,B) pseudo-first-order and second order model at pH 2; (C,D) pseudo-first-order and second order model at pH 3; (D,E) pseudo-first-order and second order model at pH 5 and (E,F) pseudo-first-order and second order model at pH 7, respectively.

Figure S16: XPS survey O 1s spectra. (1) WS; (2) MNP-BC; (3) MNPs; (4) Cr-sorbed MNP-BC; and (5) Cr-sorbed MNPs

Figure S17: FT-IR spectra of MNPs, MNP-BC and BC with, and without, Cr-sorption.

Figure S18. XRF map of Cr distribution onto MNP-BC: (A,B) pH 5 and (C,D) pH 7.

Figure S19. Correlation matrix of Fe in MNP-BC with adsorbed Cr at different pH conditions. (A) pH 5 and (B) pH 7.

Figure S20: Cr 2p XPS spectra of Cr(VI) loaded (A) MNPs and (B) MNP-BC.

Figure S21: The projection of XANES modeled Cr_2_FeO_4_ structure along the crystallography “c” axis and a 3D view of structure formed on the surface of MNPs and MNP-BC.

Figure S22: XRD patterns of MNPs and MNP-BC over time with or without Cr sorption. (A) Pure MNPs; (B) MNP-BC; (C) 2 mM Fe + 1 gL^-1^ BC + 170 µM Cr(VI) at t = 2 days; (D) 2 mM Fe + 1 gL^-1^ BC + 170 µM Cr(VI) at t = 0; (E) 10 mM Fe + 0.5 gL^-1^ BC + 350 µM Cr(VI) at t = 4 days; (F) 10 mM Fe + 0.5 gL^-1^ BC + 350 µM Cr(VI) at t = 0; (G) 10 mM Fe + 350 µM Cr(VI) at t = 4 days and (H) 10 mM Fe + 350 µM Cr(VI) at t = 0 days. The time with Cr samples represents reaction time.

Figure S23. Fe 2p XPS spectra of MNPs and MNP-BC with or without Cr sorption. (A) Pure MNPs; (B) Cr-loaded MNPs; (C) Pure MNP-BC and (D) Cr-loaded MNP-BC.

Figure 24: Fe K-edge XANES spectra of Fe references and Cr-loaded MNP-BC and MNPs. **(1)** Goethite standard; **(2)** Ferrihydrite standard; **(3)** 4 mM Fe + 1 gL^-1^ BC + 350 µM Cr(VI) at t = 4 days; **(4)** 4 mM Fe + 1 gL^-1^ BC + 350 µM Cr(VI) at t = 0; **(5)** 10 mM Fe + 350 µM at t = 4 days; **(6)** 10 mM Fe + 350 µM at t = 0; **(7)** 10 mM Fe + 1 gL^-1^ BC + 350 µM Cr(VI) at t = 4 days; **(8)** 10 mM Fe + 1 gL^-1^ BC + 350 µM Cr(VI) at t = 0 and **(**9**)** Magnetite standard. Experiments were conducted at pH 7. The time with Cr samples represents reaction time.

Figure S25: Fe K-edge EXAFS signals weighted by (A) the radial distribution function of Cr-loaded samples and (B) k^3^ spectra. (1) 4 mM Fe + 1 gL-^1^ BC + 350 µM Cr(VI) at t = 4 days; (2) 4 mM Fe + 1 gL-^1^ BC + 350 µM Cr(VI) at t = 0; (3) 10 mM Fe + 350 µM at t = 4 days; (4) 10 mM Fe + 350 µM at t = 0; (5) 10 mM Fe + 1 gL^-1^ BC + 350 µM Cr(VI) at t = 4 days and (6) 10 mM Fe + 1 gL^-1^ BC + 350 µM Cr(VI) at t = 0. Experiments were conducted at pH 7. The time with Cr samples represents reaction time.

Figure S26: Mössbauer spectroscopy of Cr-loaded MNP-BC (2 mM Fe + 1gL^-1^ BC + 170 µM Cr(VI)) at different reaction time with Cr(VI). (A) t = 0 and (B) t = 1 day at pH 7. Raw data (black dots); Sum of all fits (black); HFD Site 1 - Oh = octahedral coordinated magnetite sextets (blue); HFD Site 2 - superparamagnetic phase (yellow); HFD Site 3 - Td = tetrahedral coordinated magnetite sextets (red); QSD Site 1 - Fe(III) phase (grey).

## List of Supplemental Figures

Table S1. Sample list of Mössbauer spectroscopy analysis.

Table S2. Elemental composition, molar ratio, BET surface area, TOC and DOC of BC.

Table S3. pKas and site concentrations of BC, MNPs and MNP-BC.

Table S4. Kinetics rate constants of Cr(VI) adsorption on BC and MNP-BC.

Table S5. The fitted parameters of intraparticle diffusion model for Cr(VI) adsorption on BC and MNP-BC.

Table S6. Kinetics rate constants of Cr(VI) reduction by BC.

Table S7. Kinetics rate constants of Cr(VI) reduction by MNP-BC and MNPs.

Table S8. The linear combination fitting (LCF) results for Cr K-edge XANES spectra of Cr-laden samples.

Table S9. R-space curve fitting results of Cr K-edge EXAFS data of Cr-laden BC, MNPs and MNP-BC.

Table S10. Fe K edge EXAFS fitting results at different reaction time with Cr(VI).

Table S11. The linear combination fitting (LCF) results for Fe K-edge XANES spectra of Cr-laden samples at different reaction time with Cr(VI).

Table S12. Fitting results of Mössbauer spectroscopy of samples 1 to 5. CS = Center shif; **ε =** quadrupole shift; **ΔE_Q_ =** quadrupole split; H = hyperfine field; HFD = hyperfine field distributions; QSD = *quadrupole* splitting distributions. Errors for site populations are denoted in brackets.

## Supporting materials and methods

## Materials

Ferric chloride hexahydrate (FeCl_3_.6H_2_O), ferrous chloride tetrahydrate (FeCl_2_.4H_2_O), ammonium hydroxide (NH_4_OH), sodium hydroxide (NaOH), hydrochloric acid (HCl), sodium chlorite (NaClO_2_), sodium nitrate (NaNO_3_), potassium dichromate (K_2_Cr_2_O_7_), potassium permanganate (KMnO_4_), 1,5-diphenylcarbohydrazide (DPC) were all of ACS grade or higher, and purchased from Fisher Scientific, Canada. To prepare stock solutions and for all experiments, 18.2 MΩ.cm water was used. A biochar (BC) produced from ground willow was used for all experiments and was obtained from the Alberta Biochar Initiative (ABI; Vegreville, Alberta, Canada). The raw feedstocks were pyrolyzed under limited oxygen conditions with a residence time of 30 minutes at 500°C to 550°C, using a prototype 1.0 batch carbonizer.

## Characterization

**Morphological analysis and X-ray diffraction (XRD)**

High-resolution transmission electron microscopy (HR-TEM) images and selected area electron diffraction (SAED) patterns were obtained by using a JEM-ARM200cF S/TEM microscope, operated at 200kV. Samples were prepared using ultrathin C film on holey carbon support films of 400 mesh on Cu (from Ted Pella, Inc., Prod No. 01824) and support films of lacey formar/carbon of 200 mesh on Cu (from from Ted Pella, Inc., Prod No. 01881-F). We took 10 µL slurry from each analyzed sample, diluted each slurry with 2ml of ethanol, and then used subsamples of each diluted slurry for the analysis.

The surface morphologies of MNPs, MNP-BC and BC were characterized using a Zeiss EVO MA 15 LaB_6_ filament scanning electron microscope (SEM). The SEM magnification ranges between 20× to 20,000×, and the resolution was approximately 100 nm. An Everhart-Thornley detector was used to collect secondary electron images, and backscattered images were collected by a Si diode detector. A Peltier-cooled 10 mm^2^ Bruker Quantax 200 Silicon drift detector X-ray spectroscopy (EDS) was used to collect semi-quantitative elemental compositions.^1,2^

An X-ray diffractometer (Rikagu Ultima IV) with a cobalt source collected data between a 2θ range of 5° to 90° was used to analyze the samples. The JADE 9.5 analysis package (KS Analytical Systems) was used to fit the resulting diffraction patterns.^1,2^

**Dynamic light scattering (DLS) and Zeta potentials measurement**

The colloidal fraction of the biochar/magnetite composites was separated by centrifugation and was analyzed by DLS/ELS. The same analysis was performed for pristine biochar, and the two results were compared.

DLS and Zeta potential was obtained using a Malvern Instrument Zetasizer Nano ZS equipped with a 633 nm laser (Westborough, Massachusetts, USA). Zeta potentials were measured in 173˚ backscatter mode at different pH, using the Smoluchowski equation.^3^

**Fourier transforms infrared spectroscopy (FT-IR)**

FT-IR measurements were carried out using a Thermo Nicolet 8700 FT-IR. The samples were diluted using KBr at a 3% sample to 97% KBr ratio. The data were collected between wavenumber ranges of 400–4000 cm^−1^ with a resolution of 4 cm^−1^. Data were baseline corrected and normalized using the Thermo Scientific OMNIC FT-IR Software.

## Elemental composition and surface area

A Carlo Erba EA1108 Elemental Analyzer was used to analyze elemental compositions, including C, N, H, S, and O for BC by combusting the sample at 1000°C.^1^ The specific surface area of BC was measured using Autosorb Quantachrome 1MP instrument, and according to the Brunauer - Emmet - Teller (BET) and the Barret - Joyner - Halender (BJH) methods.

**X-ray photoelectron spectroscopy (XPS)**

XPS measurements were carried out using a Kratos Axis 165 instrument with a monochromatized Al Kα source (hν=1486.6 ev) at 12 mA and 14kV. The base pressure in the sample analytical camber (SAC) was lower than 1 x 10^-9^ torr. The pass energy was 20 eV and the step was 0.1 eV with a dwell time of 200 ms.

**Ferrozine Analysis**

Dissolved ferrous iron, Fe^2+^, reacted with ferrozine (Na_2_-3-/2-pyridyl)-5-6-bis (4-phenylsulfonate)-1,2,3-triazine) and formed an intensively purple-coloured complex. This complex was quantified spectrophotometrically at 562 nm using the cuvette photometer in Thermo Scientific Evolution 60S UV-vis Spectrophotometer. The Fe(III) content is determined as difference between Fe(tot) and Fe(II). The total Fe-content was quantified by reduction of the Fe using hydroxylamine hydrochloride.

**Cr(VI) Adsorption and Reduction Experiments**

To initiate an experiment, the sorbent was introduced into a 100 mL serum bottle and mixed with ultrapure water. After adjusting the mixture to the desired pH by adding small aliquots of NaOH or HCl, the serum bottles were sealed airtight with rubber stoppers, and the contents bubbled with N_2_ for 1 h to remove O_2_ and CO_2_ from the head space and the solution. Aliquots from the 3.5 mM Cr(VI) stock solution, prepared from K_2_Cr_2_O_7_, were injected to the serum bottle using sterile syringe to achieve the desired Cr(VI) concentration. The serum bottles were then placed on a shaker (New Brunswick Scientific Excella E5 Plataform shaker) at 200 rpm. In order to capture the initial adsorption and reduction kinetics, frequent sampling was carried out for the first 24 h: at 0, 5 min, 15 min, 0.5 h, 1 h, 2 h, 4 h, 8 h, 12 h and 24 h, followed by daily sampling for 10 days thereafter. Experiments were conducted in duplicate, and aliquots were taken from the serum bottles for both Cr(VI) reduction and adsorption analyses. To determine the Cr(VI) reduction, 500 μL of the slurry in the serum bottle was removed, mixed with 500 μL 1 M NaOH (1:1 volume ratio) in order to desorb Cr(VI) from the surface of the solids.^4^ This method has been shown to desorb Cr(VI) while leaving reduced Cr(III) species attached to the solid surface.^4-5^ To calculate the adsorption, the samples were collected from serum bottles in an identical manner, but were not mixed with NaOH. Both reduction and adsorption aliquots were filtered through 0.2 μm nylon membranes. The concentrations of Cr(VI) in the resulting filtrates were measured using the 1,5-diphenylcarbohydrazide (DPC) spectrophotometric method^6^ on a Thermo Scientific Evolution 60S UV-vis Spectrophotometer (λ_max_ = 540 nm). Total aqueous Cr was measured using Inductively Coupled Plasma - Mass Spectroscopy (ICP-MS; Agilent 8800). The concentration of Cr(III) in solution and potentially precipitated as solid was calculated by the subtraction of Cr(VI) from total Cr.

To test the effects of oxidants on the kinetics of Cr(VI) reduction by MNP-BC, 1 mg L^-1^ potassium permanganate (KMnO_4_), sodium chlorite (NaClO_2_) and sodium hypochlorite (NaClO), respectively, were injected into 100 mL serum bottles containing 10 mM Fe + 0.5 g L^-1^ BC, and spiked with 350 µM Cr(VI). The effect of dissolved oxygen (DO) was tested by purging 10 mM Fe + 0.5 g L^-1^ BC containing 350 µM Cr(VI), for two hours with a 23.5% O_2_ + 76.5% N_2_ gas mixture. These experiments were conducted at pH 7, and the experimental procedures and samples analyses were carried out as described above for Cr(VI) reduction experiments.

**Adsorption Modeling**

The equilibrium adsorption capacity (*Q_e_*, mg/g) for Cr(VI) was calculated using Eq. (1),

$Q_{e}=\frac{(C_{0}-C)\times V}{m}$ (1)

where C_0_ (mgL^-1^) and C (mgL^-1^) are the initial and equilibrium concentrations of Cr(VI) in solution after adsorption, respectively. The parameters of m(g) and V(mL) are the mass of adsorbents and the volume of the suspension, respectively.

Reaction kinetics were assessed using pseudo-first order kinetics, pseudo-second order kinetics and intra-particle diffusion models. The pseudo-first order adsorption kinetics was calculated using Eq. (2)^7^

$Log\left( Q_{e}-Q_{t} \right)=k_{1}t$ (2)

where, *k_1_* is the rate constant of pseudo-first order model (min^-1^), t is time (min), *Q_e_* is the adsorption capacity at adsorption equilibrium (mg g^-1^) and *Q_t_* is the adsorption capacity at a specific reaction time, respectively.

The pseudo-second order adsorption kinetics was calculated using Eq. (3)

$\frac{dQ_{t}}{dt}=k_{p}(Q_{e}-Q_{t})^{2}$ (3)

where, *k_p_* is the rate constant of pseudo-second order model (mg g^-1^•min^-1^), *Q_e_* is the adsorption capacity at adsorption equilibrium (mg g^-1^) and *Q_t_* is the adsorption capacity at a specific reaction time, respectively.

The intraparticle diffusion rate was calculated using^7^:

$Q_{t}=k_{id}t_{1/2}+c$ (4)

where, *k_id_* is the intraparticle diffusion rate constant, t_1/2_, is the time needed for the initial concentration of the reactant to decrease by half and *c* is the intercept.

The Chi-square test was also used to determine the best-fitting kinetics model (Table S4).

**Solid Phase Analyses after Adsorption and Reduction**

Cr K-edge (5989 eV) XAS measurements were conducted using a double Si (111) crystal monochromator detuned to 40% of the maximal intensity at the end of the EXAFS scan to reduce the influence of higher-order harmonics. During the experiment, the beamline initial X-ray energy calibration was performed by using a metallic Cr foil provided by Exafs Materials (<http://exafsmaterials.com/>). The same Cr foil was further set downstream of the detected sample throughout the entire EXAFS experiment. Thus in-step energy calibration was available for each individual EXAFS scan. Data for the reference materials (see below) were collected in transmission mode, while absorption spectra for samples were collected in fluorescence mode using a 32-element solid state Ge detector. Based on the spectral resolution desired, the scanning rate was usually 1s/point for the pre-edge and XANES data region, and 1 to 10 s/point for the EXAFS data region. Therefore, a full EXAFS spectrum often took up to 60 minutes to collect. The Cr standards included K_2_Cr_2_O_7_, Cr(III)-acetate and Cr(OH)_3._ Spectra were also collected at the Fe K-edge (7112 eV) for Cr-loaded MNPs and MNP-BC samples, to determine the transformation of MNPs to other oxidized phases of Fe mineral and coordination with Cr. Fe reference materials analyzed include magnetite, ferrihydrite and goethite. X-ray absorption near edge structure (XANES) data were processed using the graphical interface ATHENA from DEMETER. ATHENA was used for processing raw data, background subtraction and linear combination fitting to determine average Cr and Fe valence states.^8-9^ WinXAS (version 2.3) was used to model k-space and Fourier Transformation (FT) plotted spectra. The theoretical phase shift and backscattering amplitude function from the crystal structure data of Cr and Fe was calculated using the FEFF 7 code.^10^

Synchrotron-based X-ray fluorescence (XRF) mapping was carried out on thin sections of Cr-loaded BC, MNPs and MNP-BC at the Very Sensitive Elemental and Structural Probe Employing Radiation beamline (VESPERS 07B2-1) at CLS. The Cr and Fe distributions in the thin sections were measured using a single-element dispersive silicon drift XRF detector (Hitachi Vortex-90EX), and resulting fluorescence data were collected using the “Pink Beam” mode, with X-ray energy between 2-30 keV during mapping. Mapping were carried out using a 3 µm step size resolution, with a dwell time of one second per pixel.

**Mössbauer spectroscopy analysis**

Five Cr containing MNP-BC samples (Table S1), were stored in the dark at -20 °C prior to analysis. For Mössbauer sample preparation, the samples were thawed until viscous and approximately 70 mg of the wet sample was transferred into plexiglas holders (1 cm²) and frozen. Frozen samples were fixed and sealed with airtight and cold-resistance Kapton tape and kept at -20 °C until measurement. Samples were transferred to the instrument (WissEL, Gmbh) and loaded inside a closed-cycle exchange gas cryostat (Janis cryogenics). All five spectra were recorded at 140 K, which is the standard temperature for measuring magnetite containing samples as it falls above the Verwey transition (~121 K), and enough to ensure superparamagnetic magnetite is fully magnetically ordered.^10^ Measurements for all five samples were collected using a constant acceleration drive system in transmission mode with a ^57^Co/Rh source and calibrated against a 7µm thick α-^57^Fe foil measured at room temperature. All spectra were analyzed using Recoil (University of Ottawa) by applying a Voigt Based Fitting (VBF) routine.^12^ The half width at half maximum (HWHM) was fixed to a value of 0.13 mm/s for all samples.

Table S1. Sample list of Mössbauer spectroscopy analyses.

| Sample Number | Sample Description |
| --- | --- |
|  |  |
| 1 | 10 mM Fe + 0.5 gL^-1^ BC + 350 µM Cr(VI) at time = 0 |
| 2 | 10 mM Fe + 0.5 gL^-1^ BC + 350 µM Cr(VI) at time = 2 days |
| 3 | 10 mM Fe + 0.5 gL^-1^ BC + 350 µM Cr(VI) at time = 4 days |
| 4 | 2 mM Fe + 1 gL^-1^ BC + 170 µM Cr(VI) at time = 0 |
| 5 | 2 mM Fe + 1 gL^-1^ BC + 170 µM Cr(VI) at time = 1 day |


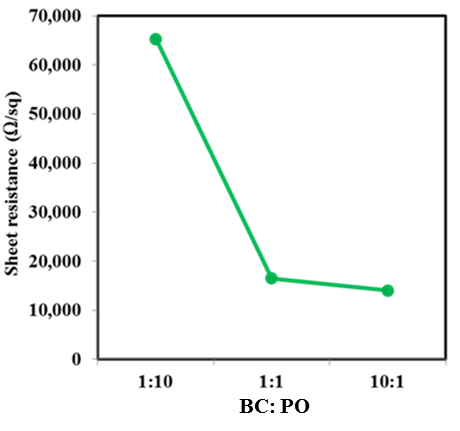


Figure S1: Sheet resistance of BC at different concentration.

^
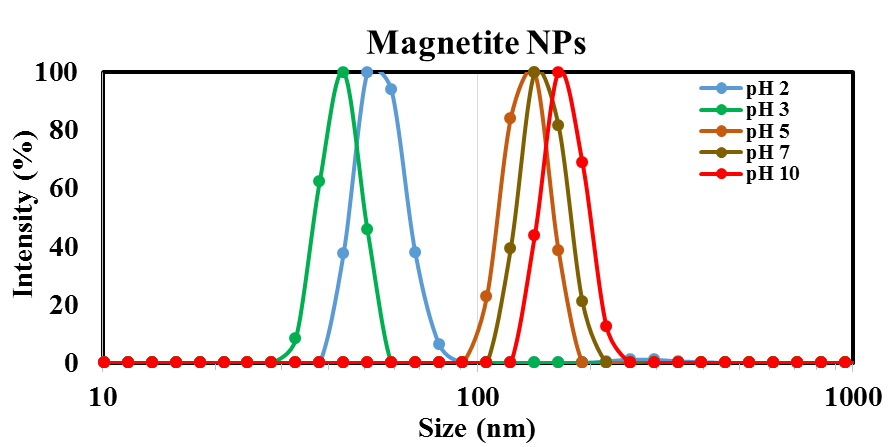
^

Figure S2: Size distribution of MNPs at tested pH conditions.


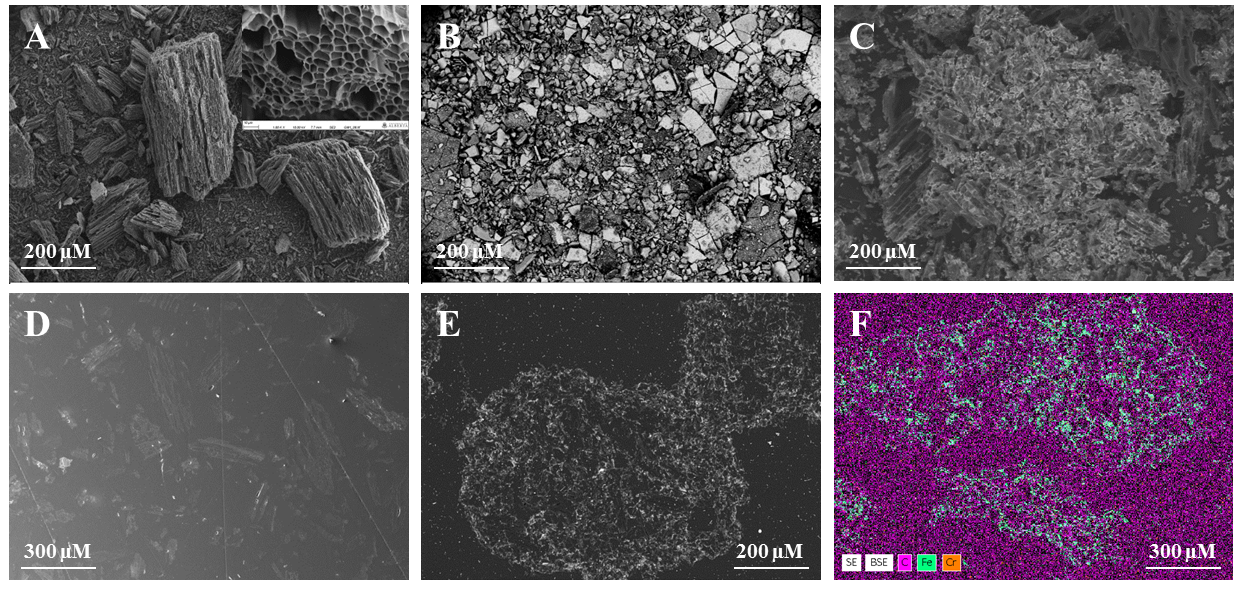


Figure S3. SEM images of (A) BC; (B) MNPs; (C) MNP-BC (high Fe concentration); (D) back scattered electron (BSE) map of BC in thin section; (E) BSE map of MNP-BC in thin section; and (F) EDS of MNP-BC.


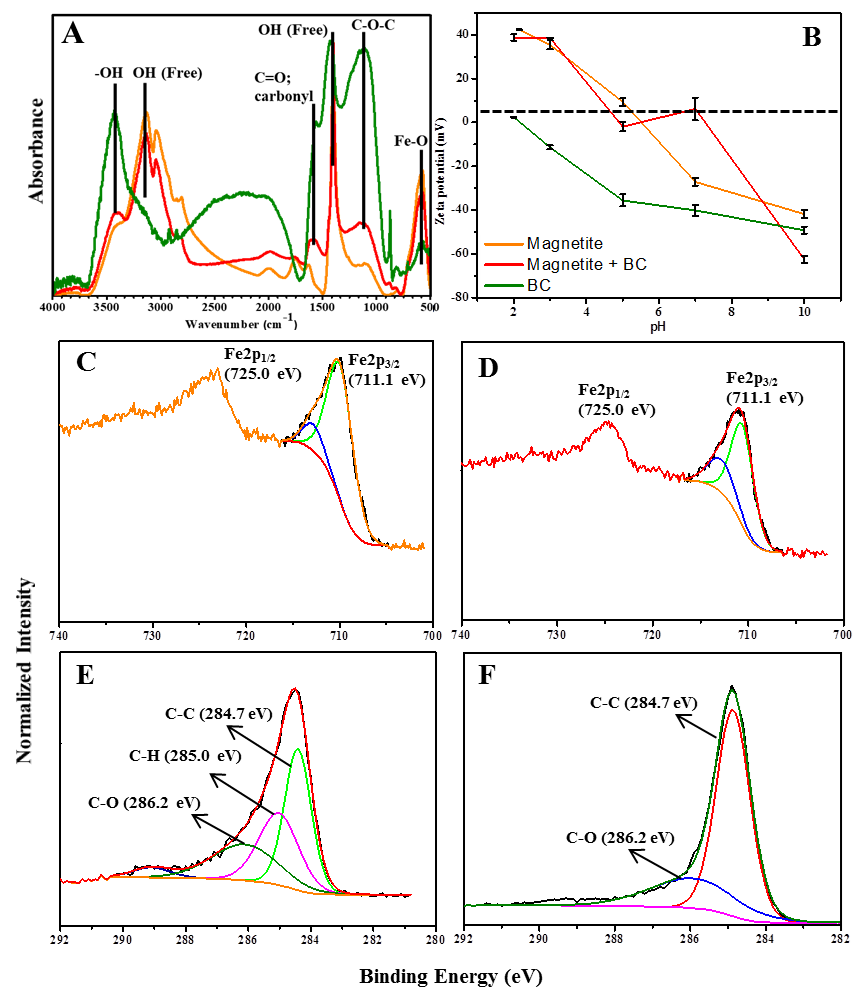


Figure S4. Spectroscopic analyses of MNPs, BC, and MNP-BC. (A) FT-IR spectra of MNPs, MNP-BC and BC; (B) Zeta potentials of MNPs, MNP-BC and BC; XPS spectra (C-F) (C) Fe 2p of MNPs; (D) Fe 2p of MNP-BC; (E) C 1s of MNP-BC and (F) C 1s of BC.


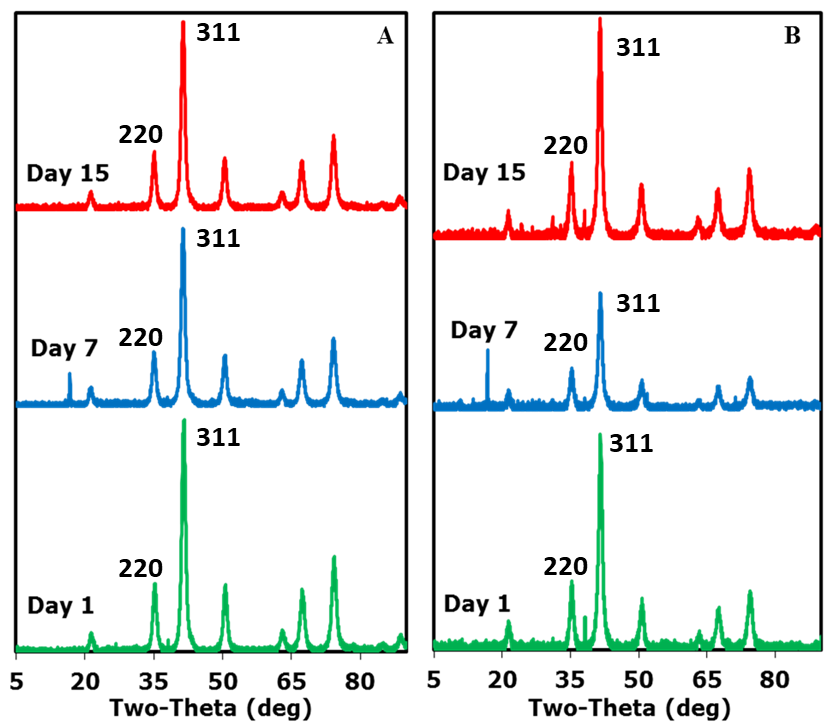


Figure S5: XRD patterns of (A) MNPs and (B) MNP-BC.


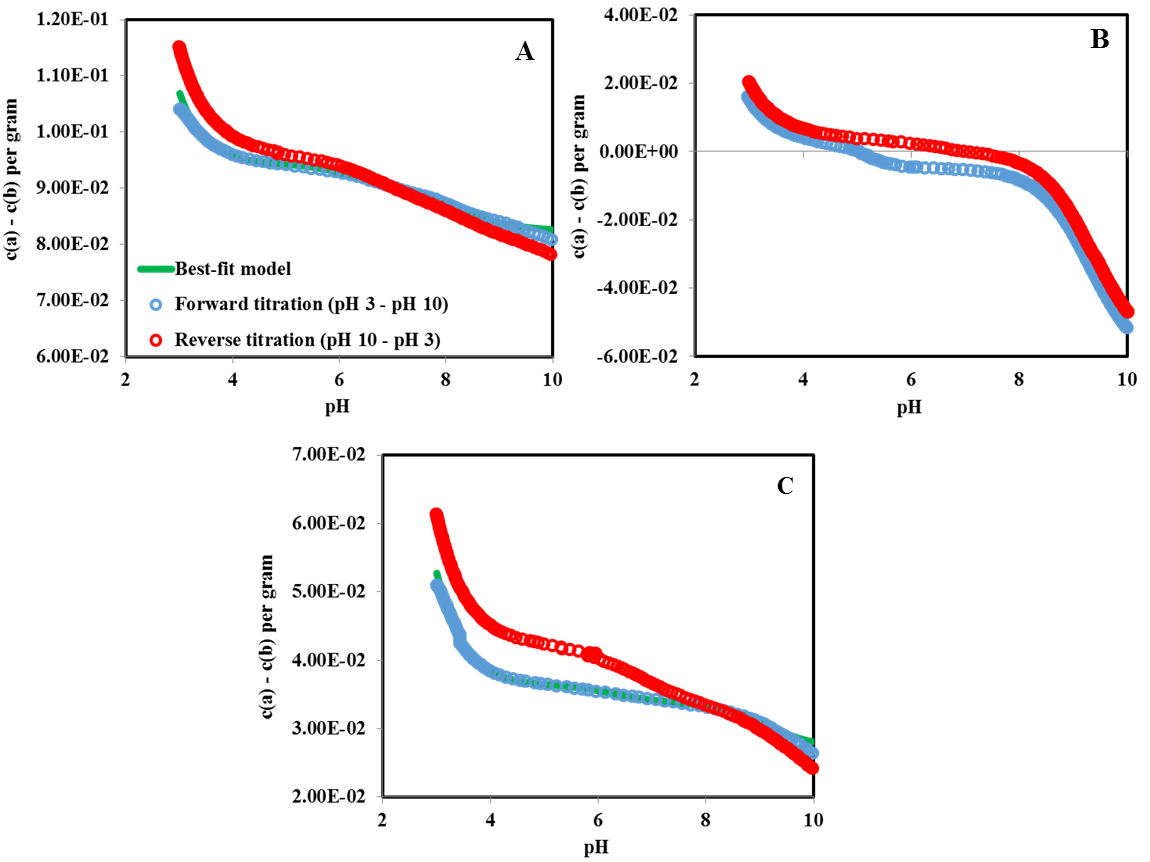


Figure S6: Potentiometric titrations of (A) BC; (B) MNPs and (C) MNP-BC.


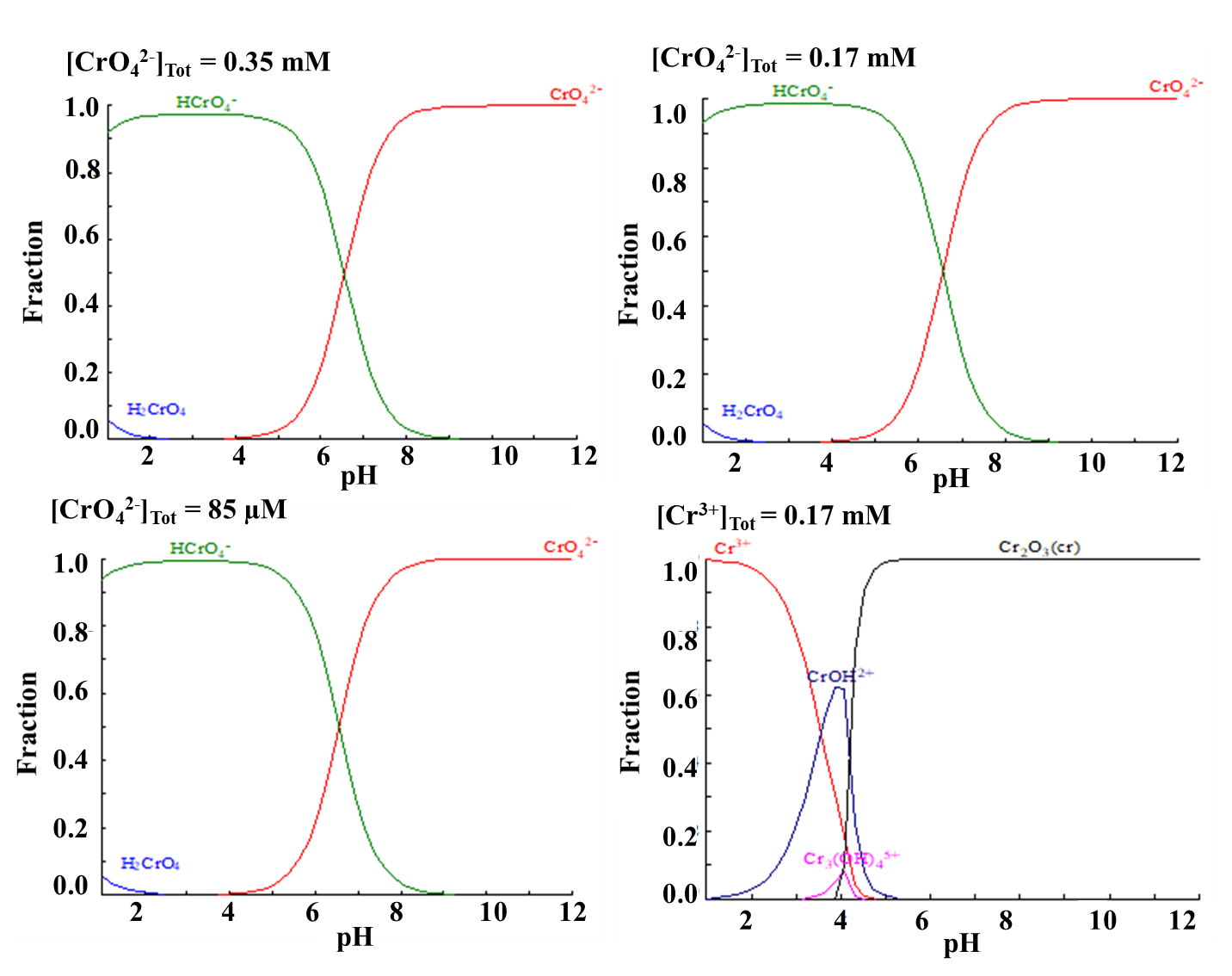


**C**

**B**

**A**

**D**

Figure S7: Speciation diagram of Cr(VI) and Cr(III). (A) 350 µM Cr(VI); (B) 170 µM Cr(VI); (C) 85 µM Cr(VI) and (D) Cr(III).

Figure S8: Zeta potentials of MNPs, MNP-BC and BC with and without Cr-sorption (n=3).


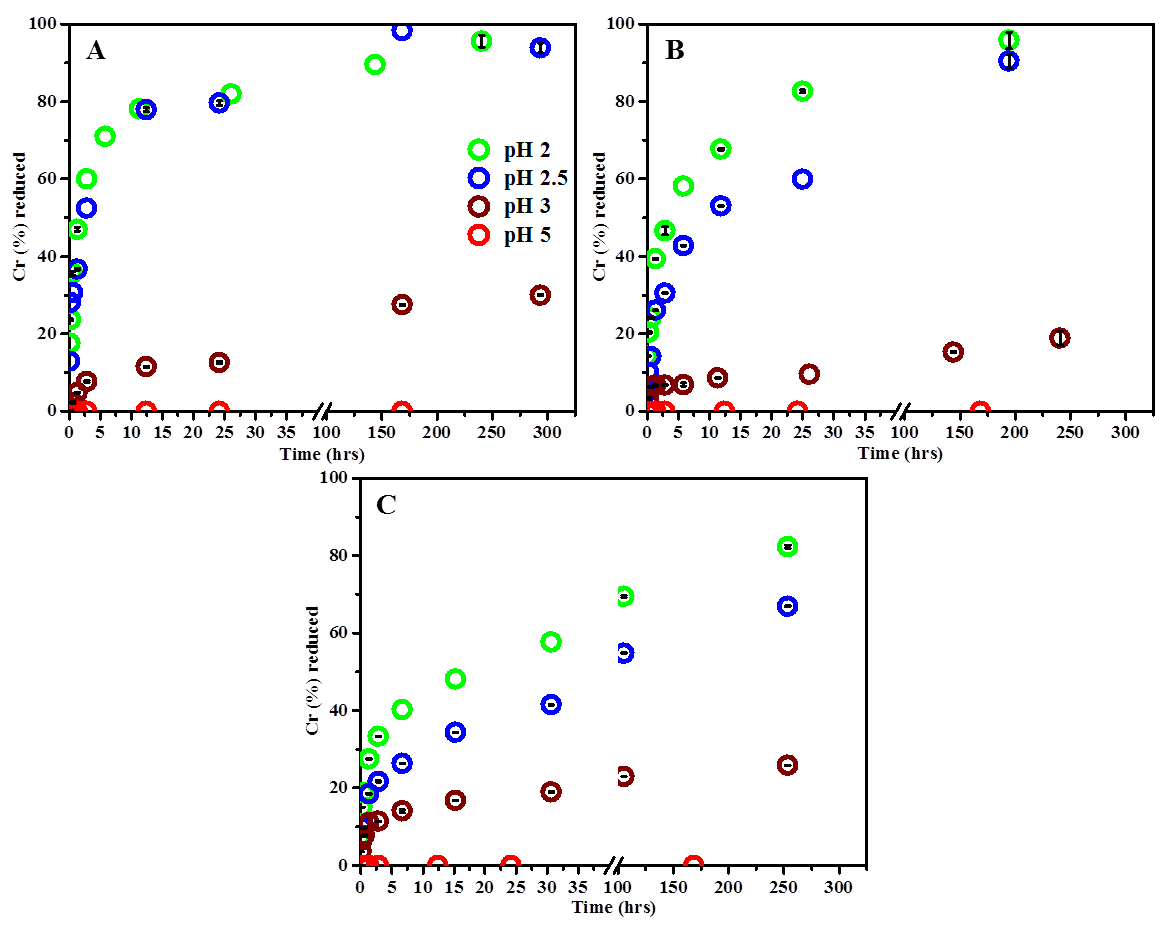


Figure S9: Cr(VI) reduction by 1 g L^-1^ BC at different pH conditions. (A) 350 µM Cr(VI); (B) 170 µM Cr(VI); and (C) 85 µM Cr(VI).

Figure S10: Reusability of the MNP-BC composites


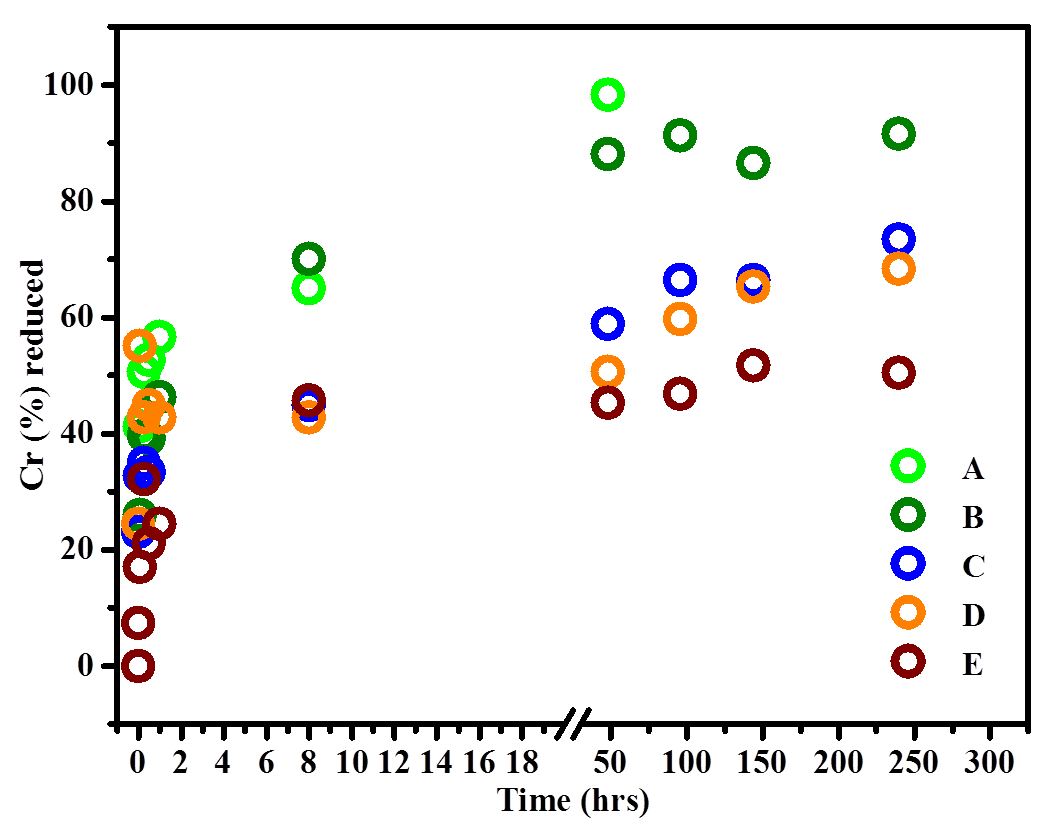


Figure S11. Effects of dissolved oxygen (DO) and selected oxidants on Cr(VI) reduction by MNP-BC. (A) 10 mM Fe + 1 gL^-1^ BC (no oxidants); (B) DO; (C) KMnO_4_; (D) NaClO_2_ and (E) NaClO.


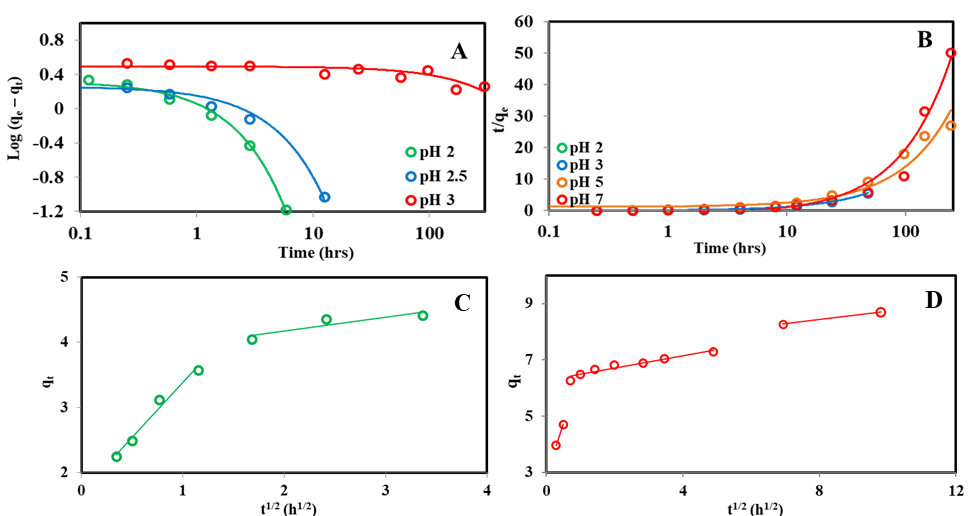


Figure S12. Adsorption kinetics and intraparticle diffusion for Cr(VI) adsorption on BC and MNP-BC. (A) Cr(VI) adsorption on BC fitted with pseudo-first-order; (B) Cr(VI) adsorption on MNP-BC fitted with pseudo-second-order models and (C, D) intraparticle diffusion plot for Cr(VI) adsorption on BC and MNP-BC, respectively.


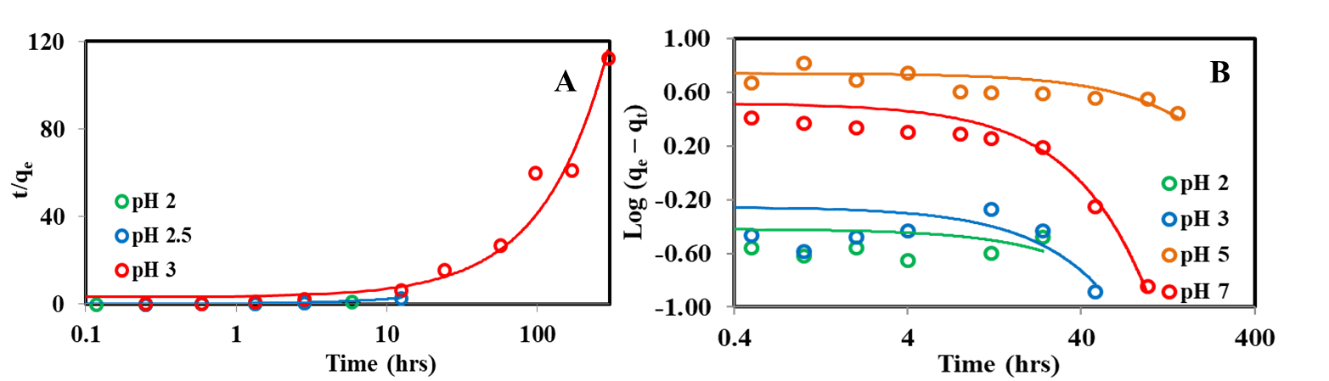


Figure S13. Adsorption kinetics for Cr(VI) adsorption on BC and MNP-BC. (A) Cr(VI) adsorption on BC fitted with pseudo-second-order, and (B) Cr(VI) adsorption on MNP-BC fitted with pseudo-first-order models.


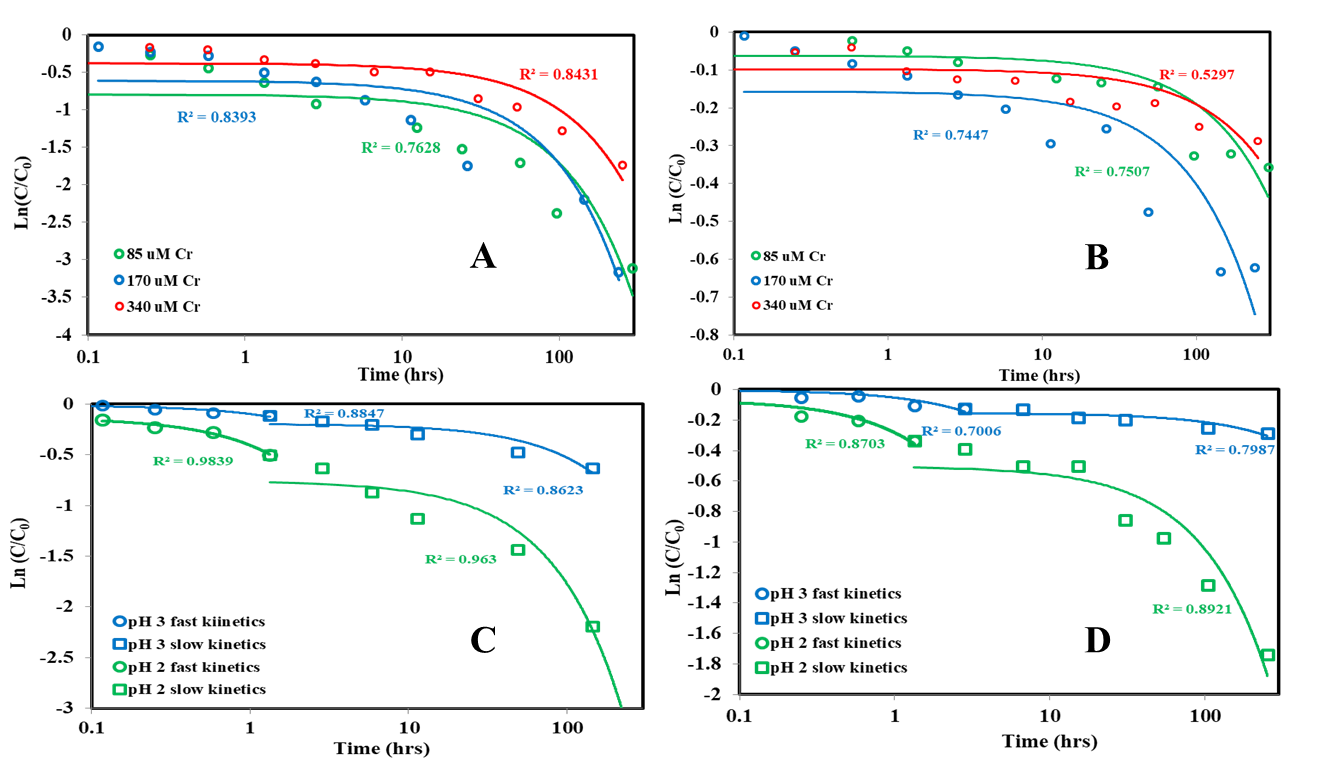


Figure S14. Reduction kinetics for Cr(VI) reduction by BC at different Cr concentrations and pH conditions. (A, B) Cr(VI) reduction on BC fitted with pseudo-first-order model at pH 2 and 3, respectively, and (C, D) Cr(VI) reduction on BC fitted with pseudo-first-order model using fast and slow kinetics at pH 2 and 3, respectively.


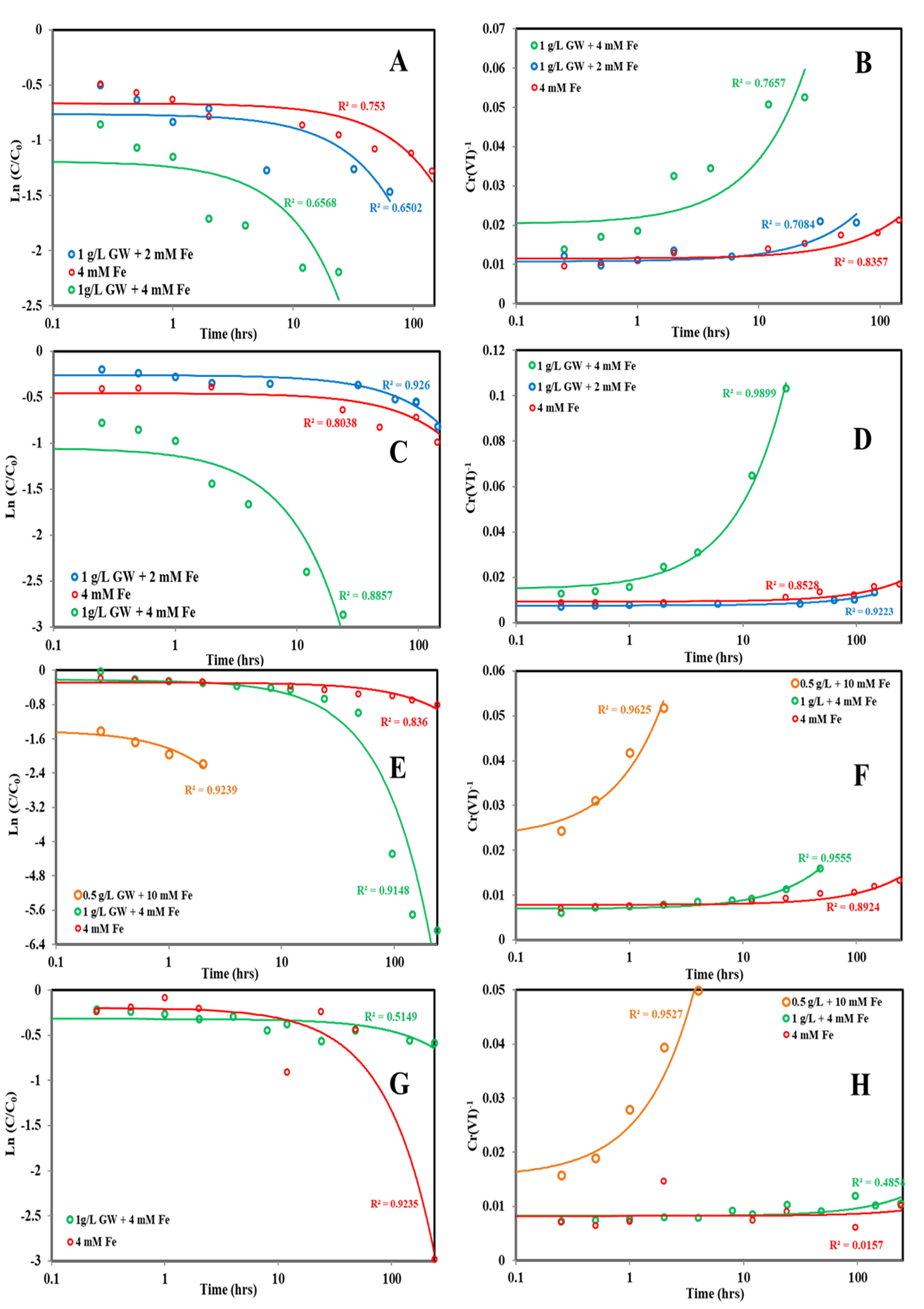


Figure S15. Reduction kinetics for Cr(VI) reduction by MNP-BC and MNPs at different concentrations and pH conditions. (A, B) pseudo-first-order and second order model at pH 2; (C, D) pseudo-first-order and second order model at pH 3; (D, E) pseudo-first-order and second order model at pH 5 and (E, F) pseudo-first-order and second order model at pH 7, respectively.


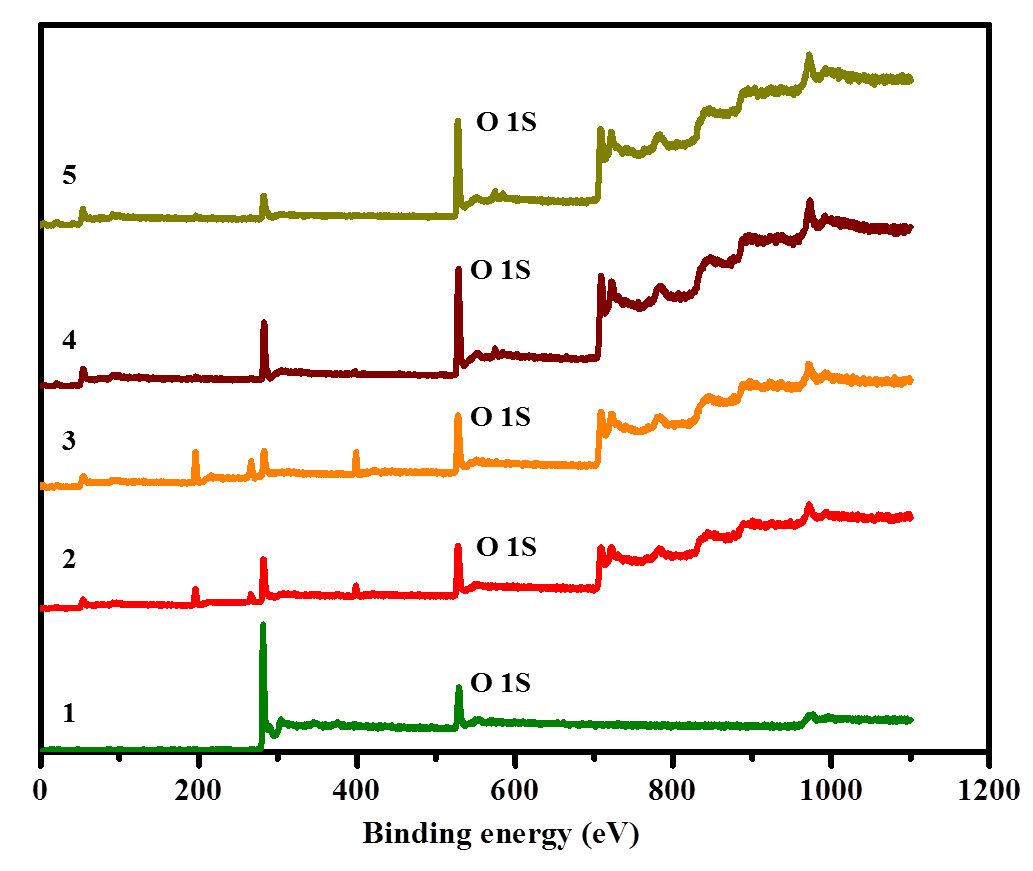


Figure S16: XPS survey O 1s spectra. (1) WS; (2) MNP-BC; (3) MNPs; (4) Cr-sorbed MNP-BC; and (5) Cr-sorbed MNPs


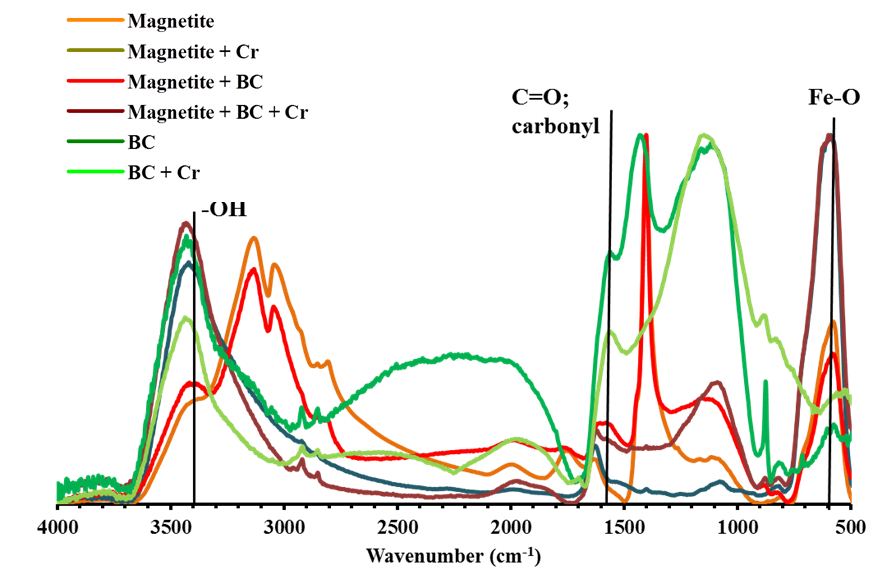


Figure S17: FTIR spectra of MNPs, MNP-BC and BC with, and without, Cr-sorption.


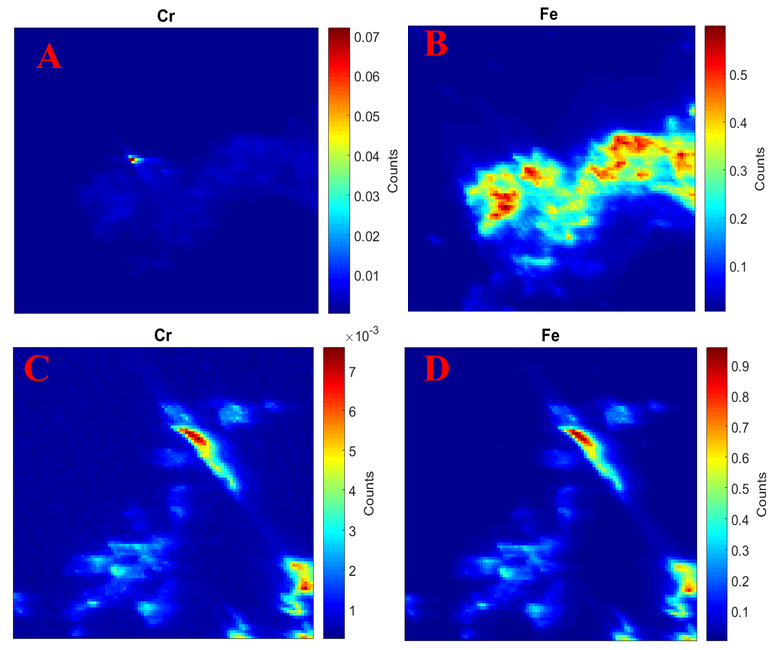


Figure S18. XRF map of Cr distribution onto MNP-BC: (A,B) pH 5 and (C,D) pH 7.

**
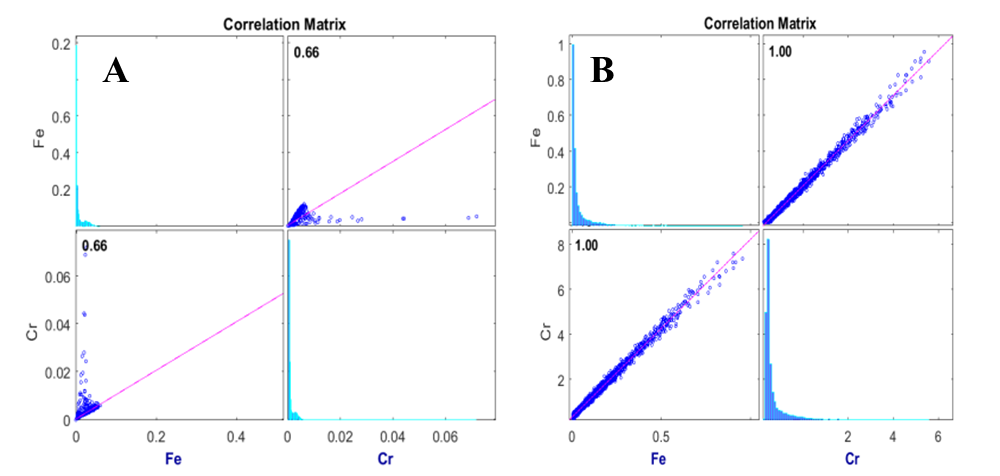
**

Figure S19. Correlation matrix of Fe in MNP-BC with adsorbed Cr at tested pH conditions. (A) pH 5 and (B) pH 7.


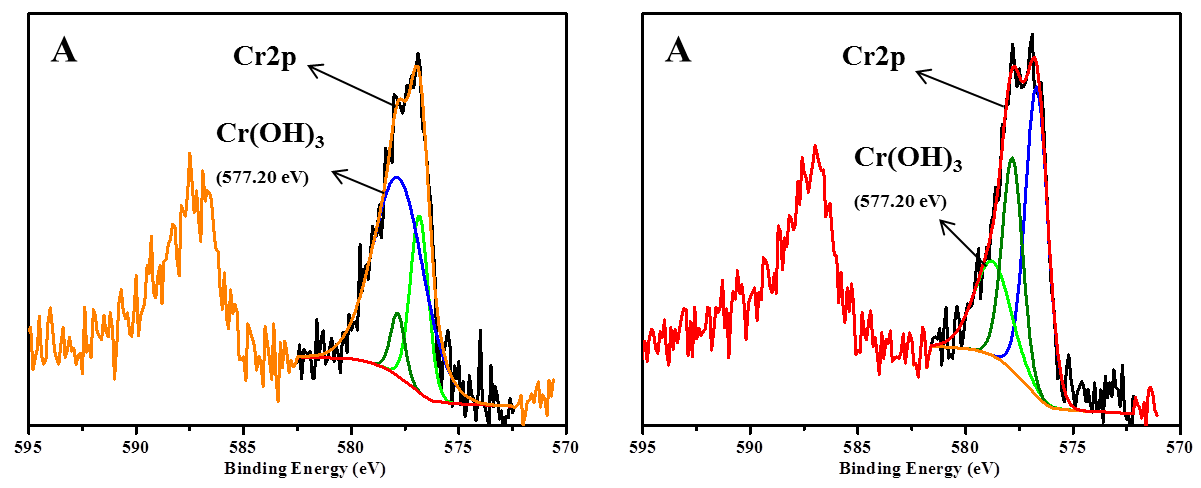


**B**

Figure S20: Cr 2p XPS spectra of Cr(VI) loaded (A) MNPs and (B) MNP-BC.


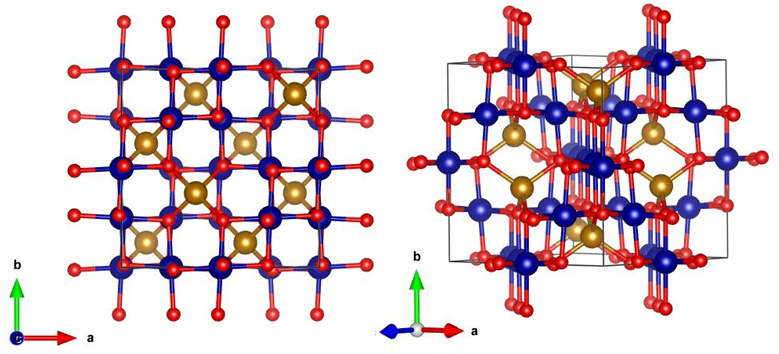


Figure S21: The projection of XANES modeled Cr_2_FeO_4_ structure along the crystallography “c” axis and a 3D view of structure formed on the surface of MNPs and MNP-BC.


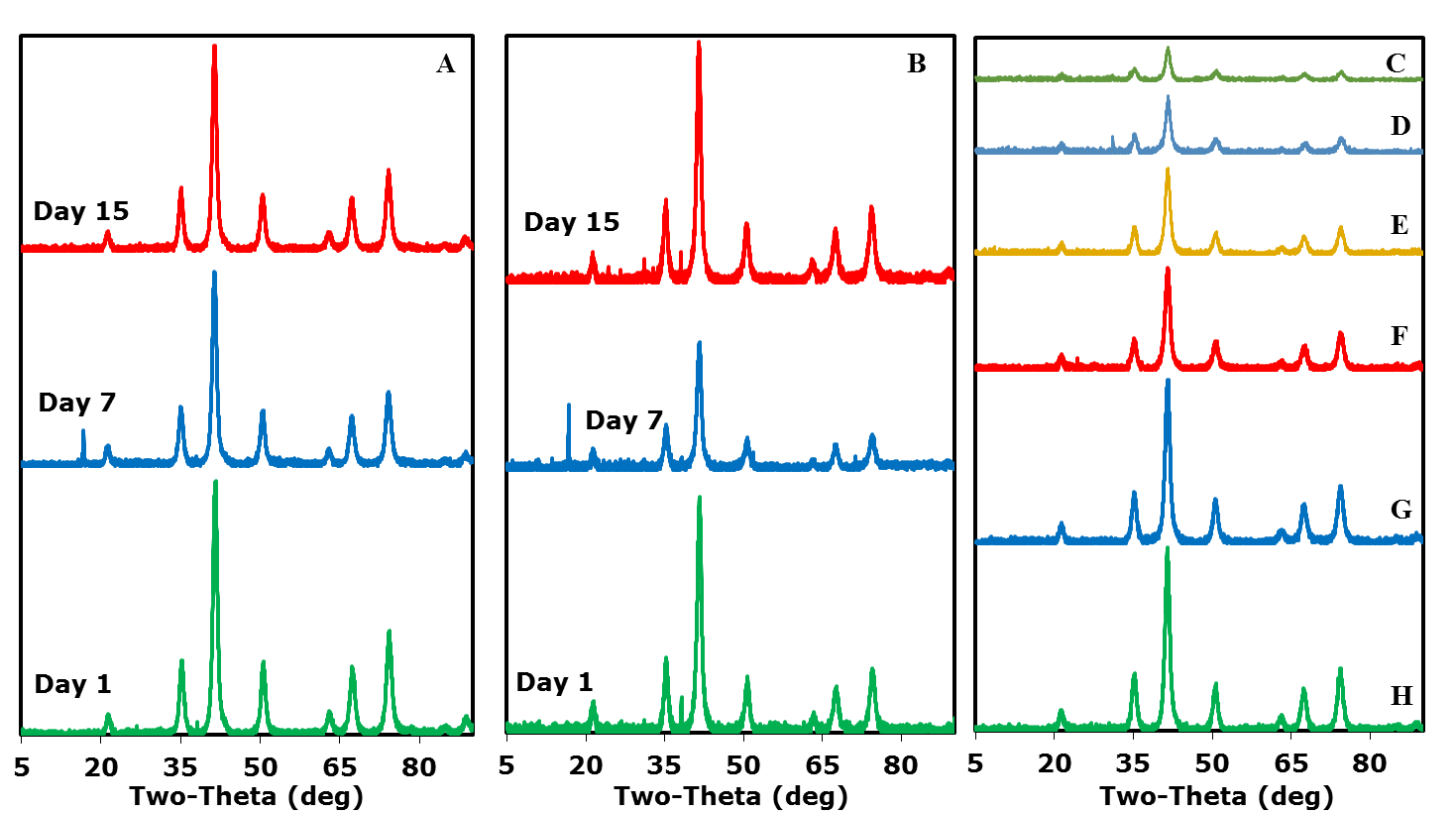


Figure S22: XRD patterns of MNPs and MNP-BC over time with or without Cr sorption. (A) Pure MNPs; (B) MNP-BC; (C) 2 mM Fe + 1 gL^-1^ BC + 170 µM Cr(VI) at t = 2 days; (D) 2 mM Fe + 1 gL^-1^ BC + 170 µM Cr(VI) at t = 0; (E) 10 mM Fe + 0.5 gL^-1^ BC + 350 µM Cr(VI) at t = 4 days; (F) 10 mM Fe + 0.5 gL^-1^ BC + 350 µM Cr(VI) at t = 0; (G) 10 mM Fe + 350 µM Cr(VI) at t = 4 days and (H) 10 mM Fe + 350 µM Cr(VI) at t = 0 days. The time with Cr samples represents reaction time.


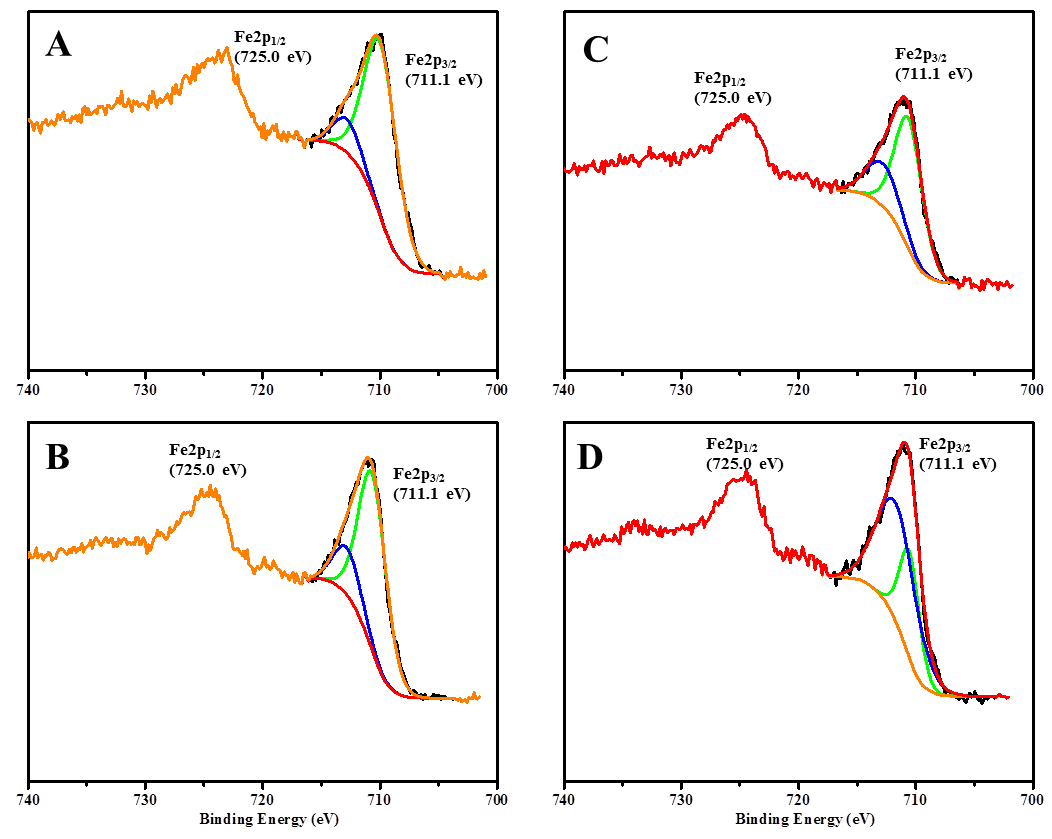


Figure S23. Fe 2p XPS spectra of MNPs and MNP-BC with or without Cr sorption. (A) Pure MNPs; (B) Cr-loaded MNPs; (C) Pure MNP-BC and (D) Cr-loaded MNP-BC.


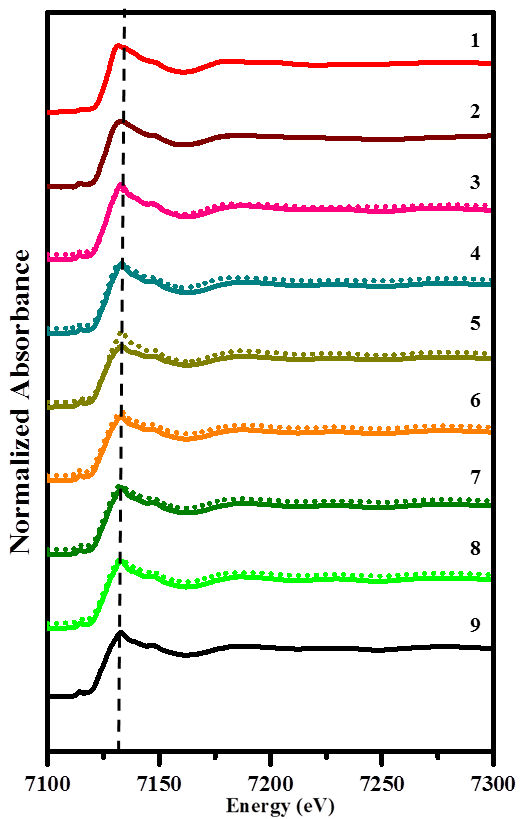


Figure 24: Fe K-edge XANES spectra of Fe references and Cr-loaded MNP-BC and MNPs. **(1)** Goethite standard; **(2)** Ferrihydrite standard; **(3)** 4 mM Fe + 1 gL^-1^ BC + 350 µM Cr(VI) at t = 4 days; **(4)** 4 mM Fe + 1 gL^-1^ BC + 350 µM Cr(VI) at t = 0; **(5)** 10 mM Fe + 350 µM at t = 4 days; **(6)** 10 mM Fe + 350 µM at t = 0; **(7)** 10 mM Fe + 1 gL^-1^ BC + 350 µM Cr(VI) at t = 4 days; **(8)** 10 mM Fe + 1 gL^-1^ BC + 350 µM Cr(VI) at t = 0 and **(**9**)** Magnetite standard. Experiments were conducted at pH 7. The time noted for each sample reflects reaction time with Cr(VI).


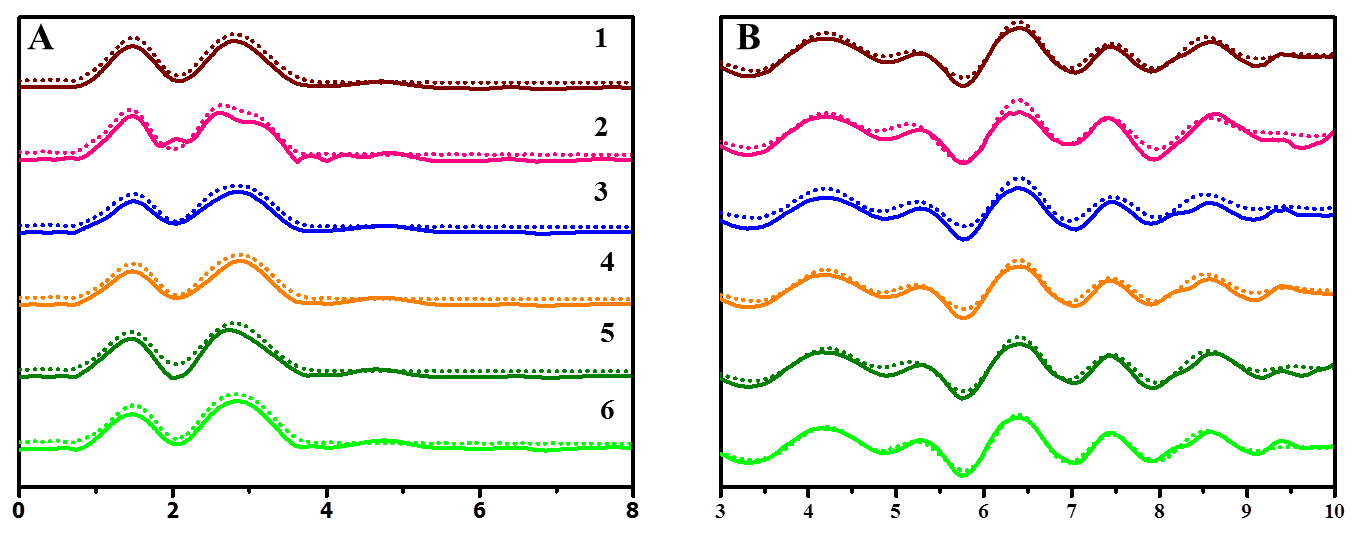


Figure S25: Fe K-edge EXAFS signals weighted by (A) the radial distribution function of Cr-loaded samples and (B) k^3^ spectra. (1) 4 mM Fe + 1 gL-^1^ BC + 350 µM Cr(VI) at t = 4 days; (2) 4 mM Fe + 1 gL-^1^ BC + 350 µM Cr(VI) at t = 0; (3) 10 mM Fe + 350 µM at t = 4 days; (4) 10 mM Fe + 350 µM at t = 0; (5) 10 mM Fe + 1 gL^-1^ BC + 350 µM Cr(VI) at t = 4 days and (6) 10 mM Fe + 1 gL^-1^ BC + 350 µM Cr(VI) at t = 0. Experiments were conducted at pH 7. The time noted for each sample reflects reaction time with Cr(VI).


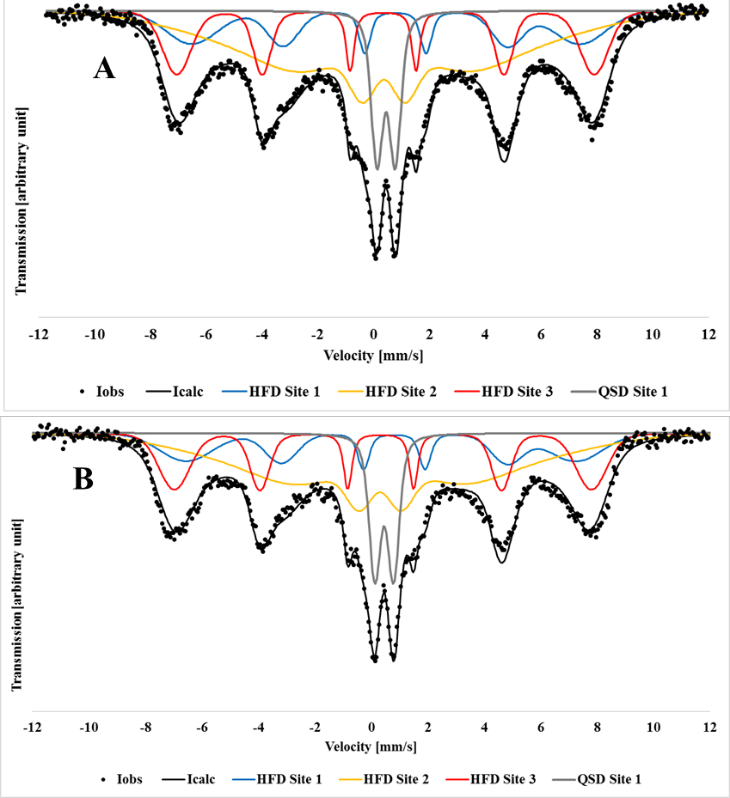


Figure S26: Mössbauer spectroscopy of Cr-loaded MNP-BC (2 mM Fe + 1gL^-1^ BC + 170 µM Cr(VI)) at different reaction time with Cr(VI). (A) t = 0 and (B) t = 1 day at pH 7. Raw data (black dots); Sum of all fits (black); HFD Site 1 - Oh = octahedral coordinated magnetite sextets (blue); HFD Site 2 - superparamagnetic phase (yellow); HFD Site 3 - Td = tetrahedral coordinated magnetite sextets (red); QSD Site 1 - Fe(III) phase (grey).

Table S2. Elemental composition, molar ratio, BET surface area, TOC and DOC of BC.

| **Properties** | **BC** | |
| --- | --- | --- |
|  |  |  |
| %C | 76.6 |  |
| %H | 1.6 |  |
| %O | 10.2 |  |
| %N | 0.6 |  |
| Molar H/C | 0.3 |  |
| Molar O/C | 0.1 |  |
| Surface area (m^2^/g) | 319 |  |
| TOC (w/w%) | 73 |  |
| DOC (mg/L) | 28 |  |

| Adsorbents | Total Sites | logK_1_ | logK_2_ | logK_3_ | Site 1 concentration (mol/g) | Site 2 concentration (mol/g) | Site 3 concentration (mol/g) |
| --- | --- | --- | --- | --- | --- | --- | --- |
| BC | 3 | 2.4 | 6.3 | 8.3 | 8.8E-04 | 4.2E-04 | 6.3E-04 |
| MNPs | 2 | 5 | 6.2 |  | 3.2E-03 |  |  |
| MNP-BC | 3 | 2.8 | 6.3 | 9.08 | 1.4E-03 | 2.5E-04 | 6.0E-04 |

Table S3. pKa values and site concentrations of BC, MNPs and MNP-BC.

Table S4. Kinetics rate constants of Cr(VI) adsorption on BC and MNP-BC.

| Materials | C_0_ | pH | Pseudo-first-order model | | χ^2^ test | Pseudo-second-order model | | χ^2^ test |
| --- | --- | --- | --- | --- | --- | --- | --- | --- |
|  |  |  | K_1_ (hr^-1^) | R^2^ |  | K_2_ (g/mg.hr) | R^2^ |  |
| BC | 85 µM | 2 | 0.28 | 0.96 | 0.08 | 0.25 | 0.99 | 0.75 |
|  |  | 2.5 | 0.11 | 0.90 | 0.9 | 0.23 | 0.99 | 0.15 |
|  |  | 3 | 0.001 | 0.70 | 1 | 0.38 | 0.96 | 10^-5^ |
| MNP-BC  (4 mM Fe + 1 gL^-1^ BC) | 170 µM | 2 | 0.006 | 0.01 | - | 0.12 | 1 | 0.14 |
|  |  | 3 | 0.02 | 0.1 | - | 0.11 | 0.99 | 0.003 |
|  |  | 5 | 0.01 | 0.86 | 1 | 0.13 | 0.93 | 0.000006 |
|  |  | 7 | 0.002 | 0.5 | 0.86 | 0.20 | 0.96 | 0.000007 |

Table S5. The fitted parameters of intraparticle diffusion model for Cr(VI) adsorption on BC and MNP-BC.

| Materials | C_0_ | pH | K_1_ (mg g-^1^hr^-0.5^) | R^2^ | K_2_ (mg g-^1^hr^-0.5^) | R^2^ | K_3_ (mg g-^1^hr^-0.5^) | R^2^ |
| --- | --- | --- | --- | --- | --- | --- | --- | --- |
| BC | 85 µM | 2 | 3 | 0.85 | 0.21 | 0.8 | NA | NA |
| MNP-BC  (4 mM Fe + 1 gL^-1^ BC) | 170 µM | 7 | 9.6 | 0.9 | 0.4 | 0.95 | 0.3 | 0.96 |

Table S6. Kinetics rate constants of Cr(VI) reduction by BC.

| Materials | C_0_ | pH | Pseudo-first-order model | | Pseudo-first-order model (fast) | | Pseudo-first-order model (slow) | | Pseudo-first-order model (fast) | | Pseudo-first-order model (slow) | |
| --- | --- | --- | --- | --- | --- | --- | --- | --- | --- | --- | --- | --- |
|  |  |  | K_1obs_ (hr^-1^) | R^2^ | K_1obs_ (hr^-1^) | R^2^ | K_2obs_ (hr^-1^) | R^2^ | K_1int_ (hr^-1^) | R^2^ | K_2int_ (hr^-1^) | R^2^ |
| BC | 85 µM | 2 | 0.0096 | 0.73 | NA | | | | | | | |
|  |  | 3 | 0.0013 | 0.75 |  |  |  |  |  |  |  |  |
|  | 170 µM | 2 | 0.012 | 0.82 | 0.27 | 0.98 | 0.01 | 0.96 | 0.5 | 1 | 0.5 | 1 |
|  |  | 3 | 0.0026 | 0.73 | 0.085 | 0.88 | 0.003 | 0.86 |  |  |  |  |
|  | 350 µM | 2 | 0.0064 | 0.82 | 0.21 | 0.87 | 0.005 | 0.89 | 0.6 | 1 | 0.9 | 1 |
|  |  | 3 | 0.001 | 0.52 | 0.05 | 0.70 | 0.006 | 0.80 |  |  |  |  |

Table S7. Kinetics rate constants of Cr(VI) reduction by MNP-BC and MNPs.

| Materials | C_0_ | pH | Second-order model | |
| --- | --- | --- | --- | --- |
|  |  |  | K_obs_ (µMhr^-1^) | R^2^ |
| 10 Fe + 0.5 gL^-1^ BC | 170 µM  170 µM  170 µM  170 µM | 5 | 0.014 | 0.96 |
|  |  | 7 | 0.0094 | 0.95 |
| 4 mM Fe + 1 gL^-1^ BC |  | 2 | 0.0018 | 0.74 |
|  |  | 3 | 0.0039 | 0.98 |
|  |  | 5 | 0.0002 | 0.95 |
|  |  | 7 | 1E-05 | 0.50 |
| 2 mM Fe + 1 gL^-1^ BC |  | 2 | 0.0002 | 0.68 |
|  |  | 3 | 4E-05 | 0.88 |
|  |  | 5 | 6E-06 | 0.20 |
|  |  | 7 | 2E-06 | 0.11 |
| 4 mM Fe |  | 2 | 8E-05 | 0.75 |
|  |  | 3 | 4E-05 | 0.86 |
|  |  | 5 | 3E-05 | 0.87 |
|  |  | 7 | 5E-06 | 0.02 |
|  |  |  |  |  |

| Samples | Reference materials’ proportion samples (%) | | |
| --- | --- | --- | --- |
|  | K_2_CrO_7_ | Cr(OH)_3_ | Cr(III)acetate |
| 1 g L^-1^ BC + 170 µM Cr(VI) at pH 2 | Nil | 85 | 15 |
| 1 g L^-1^ BC + 170 µM Cr(VI) at pH 3 | 10 | 88 | 2 |
| 10 mM Fe + 350 µM Cr(VI) at pH 5 | Nil | 100 | Nil |
| 10 mM Fe + 350 µM Cr(VI) at pH 7 | Nil | 100 | Nil |
| 10 Fe + 0.5 gL^-1^ BC + 350 µM Cr(VI) at pH 5 | Nil | 100 | Nil |
| 10 Fe + 0.5 gL^-1^ BC + 350 µM Cr(VI) at pH 7 | Nil | 100 | Nil |
| 4 Fe + 1 gL^-1^ BC + 350 µM Cr(VI) at pH 5 | 12 | 82 | 6 |
| 2 Fe + 1 gL^-1^ BC + 350 µM Cr(VI) at pH 7 | Nil | 92 | 8 |

Table S8. Linear combination fitting (LCF) results for Cr K-edge XANES spectra of Cr-laden samples.

Table S9. R-space curve fitting results of Cr K-edge EXAFS data of Cr-laden BC, MNPs and MNP-BC.

| **Samples** | **Paths** | **CN** | **R(Å)** | **σ^2^ (Å^2^)** |
| --- | --- | --- | --- | --- |
| 1 g L^-1^ BC at pH 2 | Cr-O | 6.0 | 2.04 | 0.005 |
|  | Cr-Cr | 3.1 | 3.10 | 0.009 |
|  | Cr-C | 4.0 | 3.15 | 0.01 |
|  | Cr-C | 8.4 | 2.38 | 0.009 |
|  | Cr-C | 3.9 | 4.13 | 0.009 |
|  | Cr-C | 2.0 | 4.41 | 0.009 |
| 1 g L^-1^ BC at pH 3 | Cr-O | 6.0 | 2.02 | 0.004 |
|  | Cr-Cr | 4.7 | 3.04 | 0.009 |
|  | Cr-C | 6.5 | 3.25 | 0.009 |
|  | Cr-C | 6.3 | 3.30 | 0.009 |
|  | Cr-C | 9.7 | 4.00 | 0.009 |
|  | Cr-C | 9.9 | 4.44 | 0.009 |
| 10 mM Fe at pH 5 | Cr-O | 6.0 | 1.99 | 0.002 |
|  | Cr-Cr | 3.9 | 3.03 | 0.005 |
|  | Cr-Fe | 1.7 | 3.53 | 0.005 |
| 10 mM Fe at pH 7 | Cr-O | 6.0 | 2.02 | 0.002 |
|  | Cr-Cr | 2.6 | 3.01 | 0.005 |
|  | Cr-Fe | 2.2 | 3.62 | 0.005 |
| 10 mM Fe + 0.5 g L^-1^ BC at pH 5 | Cr-O | 6.0 | 2.02 | 0.002 |
|  | Cr-Cr | 3.7 | 3.03 | 0.005 |
|  | Cr-C | 10.0 | 3.15 | 0.005 |
|  | Cr-Fe | 1.9 | 3.55 | 0.006 |
| 4 mM Fe + 1 g L^-1^ BC at pH 5 | Cr-O | 6.0 | 2.02 | 0.003 |
|  | Cr-Cr | 0.4 | 2.93 | 0.005 |
|  | Cr-C | 5.6 | 3.08 | 0.005 |
|  | Cr-Fe | 0.8 | 3.54 | 0.006 |
| 2 mM Fe + 1 g L^-1^ BC at pH 7 | Cr-O | 6.0 | 1.97 | 0.003 |
|  | Cr-Cr | 0.9 | 2.98 | 0.004 |
|  | Cr-C | 5.0 | 3.06 | 0.005 |
|  | Cr-Fe | 0.7 | 3.51 | 0.006 |

Table S10. Fe K edge EXAFS fitting results at different reaction time with Cr(VI).

| **Samples** | **Paths** | **CN** | **R(Å)** | **σ^2^ (Å^2^)** |
| --- | --- | --- | --- | --- |
| 10 mM Fe + 0.5 gL^-1^ BC + 350 µM Cr(VI) at t = 0 | Fe-O | 4.5 | 1.94 | 0.009 |
|  | Fe-Fe | 3.0 | 2.99 | 0.008 |
|  | Fe-Fe | 5.4 | 3.46 | 0.009 |
|  | Fe-O | 3.3 | 3.53 | 0.01 |
| 10 mM Fe + 0.5 gL^-1^ BC + 350 µM Cr(VI) at t = 2 days | Fe-O | 4.5 | 1.92 | 0.009 |
|  | Fe-Fe | 3.0 | 2.99 | 0.008 |
|  | Fe-Fe | 4.9 | 3.45 | 0.009 |
|  | Fe-O | 2.5 | 3.52 | 0.001 |
| 10 mM Fe + 350 µM Cr(VI) at t = 0 | Fe-O | 4.1 | 1.94 | 0.009 |
|  | Fe-Fe | 2.2 | 2.99 | 0.008 |
|  | Fe-Fe | 5.1 | 3.46 | 0.009 |
|  | Fe-O | 3.8 | 3.53 | 0.001 |
| 10 mM Fe + 350 µM Cr(VI) at t = 2 days | Fe-O | 3.9 | 1.95 | 0.0097 |
|  | Fe-Fe | 2.4 | 2.97 | 0.008 |
|  | Fe-Fe | 5.0 | 3.45 | 0.0085 |
|  | Fe-O | 3.0 | 3.53 | 0.01 |
| 4 mM Fe + 1 gL^-1^ BC + 350 µM Cr(VI) at t = 0 | Fe-O | 5.1 | 1.91 | 0.009 |
|  | Fe-Fe | 3.7 | 2.97 | 0.008 |
|  | Fe-Fe | 5.3 | 3.46 | 0.009 |
|  | Fe-O | 1.2 | 3.52 | 0.01 |
| 4 mM Fe + 1 gL^-1^ BC + 350 µM Cr(VI) at t = 2 days | Fe-O | 5.1 | 1.95 | 0.009 |
|  | Fe-Fe | 2.8 | 2.99 | 0.008 |
|  | Fe-Fe | 5.7 | 3.46 | 0.008 |
|  | Fe-O | 4.7 | 3.58 | 0.01 |

Table S11. The linear combination fitting (LCF) results for Fe K-edge XANES spectra of Cr-laden samples at tested reaction times with Cr(VI).

| Samples | Reference materials’ proportion samples (%) | | |
| --- | --- | --- | --- |
|  | Magnetite | Ferrihydrite | Goethite |
| 4 mM Fe + 1 gL^-1^ BC + 350 µM Cr(VI) at t = 4 days | 40 | Nil | 60 |
| 4 mM Fe + 1 gL^-1^ BC + 350 µM Cr(VI) at t = 0 | 100 | Nil | Nil |
| 10 mM Fe + 350 µM Cr(VI) at t = 4 days | 100 | Nil | Nil |
| 10 mM Fe + 350 µM Cr(VI) at t = 0 | 100 | Nil | Nil |
| 10 mM Fe + 1 gL^-1^ BC + 350 µM Cr(VI) at t = 4 days | 70 | 10 | 20 |
| 10 mM Fe + 1 gL^-1^ BC + 350 µM Cr(VI) at t = 0 | 100 | Nil | Nil |

Table S12. Fitting results of Mössbauer spectroscopy of samples 1 to 5. CS = Center shif; **ε =** quadrupole shift; **ΔE_Q_ =** quadrupole split; H = hyperfine field; HFD = hyperfine field distributions; QSD = *quadrupole* splitting distributions. Errors for site populations are denoted in brackets.

| **Sample** |  | **Site Populations** | **CS** | **ε** | **ΔE_Q_** | **H** | **stdev(\|H\|)** | **stdev(\|ΔE_Q_\|)** |
| --- | --- | --- | --- | --- | --- | --- | --- | --- |
|  |  | (%) | (mm/s) | (mm/s) |  | (T) | (T) |  |
| **1** | HFD site 1 | 41.4(16) | 0.37 | 0.07 | - | 44.83 | 4.22 | - |
|  | HFD site 2 | 32.6(15) | 0.66 | -0.40 | - | 37.56 | 10.88 | - |
|  | HFD site 3 | 22.8(15) | 0.46 | -0.06 | - | 48.03 | 1.85 | - |
|  | QSD site 1 | 3.24(27) | 0.27 | - | 1.25 | - | - | 0.24 |
| **2** | HFD site 1 | 41.0(20) | 0.36 | 0.06 | - | 44.49 | 4.31 | - |
|  | HFD site 2 | 31.7(17) | 0.71 | -0.38 | - | 36.04 | 11.72 | - |
|  | HFD site 3 | 24.2(19) | 0.47 | -0.06 | - | 47.84 | 2.07 | - |
|  | QSD site 1 | 3.14(37) | 0.30 | - | 1.21 | - | - | 0.29 |
| **3** | HFD site 1 | 30.7(25) | 0.35 | 0.10 | - | 47.07 | 3.31 | - |
|  | HFD site 2 | 37.8(24) | 0.62 | -0.08 | - | 43.85 | 5.86 | - |
|  | HFD site 3 | 31.4(23) | 0.44 | -0.03 | - | 49.34 | 1.56 | - |
| **4** | HFD site 1 | 18.6(18) | 0.61 | -0.17 | - | 43.14 | 5.39 | - |
|  | HFD site 2 | 49.4(22) | 0.38 | 0.02 | - | 29.25 | 15.70 | - |
|  | HFD site 3 | 20.6(13) | 0.38 | 0.04 | - | 46.40 | 2.81 | - |
|  | QSD site 1 | 11.3(63) | 0.44 | - | 0.66 |  |  | 0.29 |
| **5** | HFD site 1 | 18.4(20) | 0.58 | -0.24 | - | 42.82 | 5.51 | - |
|  | HFD site 2 | 47.0(25) | 0.35 | 0.04 | - | 28.69 | 15.53 | - |
|  | HFD site 3 | 22.4(16) | 0.37 | 0.04 | - | 45.82 | 3.05 | - |
|  | QSD site 1 | 12.3(75) | 0.46 | - | 0.66 | - | - | 0.29 |

**References**

1. Alam, M. S., Cossio, M., Robinson, L., Kenney, J. P. L., Wang, X., Konhauser, K. O., MacKenzie, M. D., Ok, Y. S., Alessi, D. S. Removal of organic acids from water using biochar and petroleum coke. *Environ. Technol. Innovat.* 2016, 6: 141-151.
2. Alam, M.S., Swaren, L, Gunten, K.V., Cossio, M., Robbins, L.J., Flynn, S.L., Konhauser, K.O, Alessi, D. S. Application of surface complexation modeling to trace metals uptake by biochar-amended agricultural soils. *App. Geochem.* 2018, 88:103-112.
3. Hunter, R. J. Zeta potential in colloid science: Principles and Applications, *Academic Press*, 1981.
4. American Public Health Association, American Water Works Association, & Water Environment Federation. Standard Methods for the Examination of Water and Wastewater. Standard Methods. 1991, 541.
5. Cossio, M. Mechanisms of the reductive immobilization of hexavalent chromium by Wheat Straw biochar. 2017. MSc Thesis. University of Alberta.
6. EPA. (1992). METHOD 7196A - Colormeric Method, (July), 1–6.
7. Wang, T., Zhang, L., Li, C., Yang, W., Song, T., Tang, C., Meng, Y., Dai, S., Wang, H., Chai, L., Luo, J. Synthesis of Core Shell Magnetic Fe_3_O_4_@poly(m-Phenylenediamine) Particles for Chromium Reduction and Adsorption. *Environ. Sci. Technol*. 2015, 49, 5654-5662.
8. Ravel B. and Newville M. [ATHENA, ARTEMIS, HEPHAESTUS: data analysis for X-ray absorption spectroscopy using IFEFFIT](http://journals.iucr.org/s/issues/2005/04/00/ph5155/index.html). J. Synchrotron Rad. 2005, **12**, pp 537-541.
9. 37. Ressler T. WinXAS: A new software package not only for the analysis of energy-dispersive XAS data. *J. de Physique IV*. 1997, **7**(C2): 269-270.
10. 38. Ankudinov, A., Rehr, J.J. Theory of solid state contributions to the x-ray elastic scattering amplitude. *Phys. Rev. B.* 2000, 62, 2437-2445.
11. Gorski, C. A. et al. Determination of nanoparticulate magnetite stoichiometry by Mössbauer spectroscopy, acidic dissolution, and powder X-ray diffraction: A critical review. *American Mineralogist*. 2010, 95, 1017-1026.
12. Rancourt D.G., and Ping, J.Y. Voigt-based methods for arbitrary-shape static hyperfine parameter distributions in Mössbauer spectroscopy. *Nuclear Instruments and Methods in Physics Research*. 1991, B58, 85-97.
